# Emergence and suppression of cooperation by action visibility in transparent games

**DOI:** 10.1371/journal.pcbi.1007588

**Published:** 2020-01-09

**Authors:** Anton M. Unakafov, Thomas Schultze, Alexander Gail, Sebastian Moeller, Igor Kagan, Stephan Eule, Fred Wolf

**Affiliations:** 1 Georg-Elias-Müller-Institute of Psychology, University of Goettingen, Goettingen, Germany; 2 Max Planck Institute for Dynamics and Self-Organization, Goettingen, Germany; 3 Leibniz ScienceCampus Primate Cognition, Goettingen, Germany; 4 Campus Institute for Dynamics of Biological Networks, Goettingen, Germany; 5 Max Planck Institute for Experimental Medicine, Goettingen, Germany; 6 German Primate Center—Leibniz Institute for Primate Research, Goettingen, Germany; 7 Bernstein Center for Computational Neuroscience, Goettingen, Germany; University of Illinois at Urbana-Champaign, UNITED STATES

## Abstract

Real-world agents, humans as well as animals, observe each other during interactions and choose their own actions taking the partners’ ongoing behaviour into account. Yet, classical game theory assumes that players act either strictly sequentially or strictly simultaneously without knowing each other’s current choices. To account for action visibility and provide a more realistic model of interactions under time constraints, we introduce a new game-theoretic setting called transparent games, where each player has a certain probability of observing the partner’s choice before deciding on its own action. By means of evolutionary simulations, we demonstrate that even a small probability of seeing the partner’s choice before one’s own decision substantially changes the evolutionary successful strategies. Action visibility enhances cooperation in an iterated coordination game, but reduces cooperation in a more competitive iterated Prisoner’s Dilemma. In both games, “Win–stay, lose–shift” and “Tit-for-tat” strategies are predominant for moderate transparency, while a “Leader-Follower” strategy emerges for high transparency. Our results have implications for studies of human and animal social behaviour, especially for the analysis of dyadic and group interactions.

## Author summary

Humans and animals constantly make social decisions. Should an animal during group foraging or a human at the buffet try to obtain an attractive food item but risk a confrontation with a dominant conspecific, or is it better to opt for a less attractive but non-confrontational choice, especially when considering that the situation will repeat in the future? To model decision-making in such situations game theory is widely used. However, classic game theory assumes that agents act either at the same time, without knowing each other’s choices, or one after another. In contrast, humans and animals usually try to take the behaviour of their opponents and partners into account, to instantaneously adjust their own actions if possible. To provide a more realistic model of decision making in a social setting, we here introduce the concept of transparent games. It integrates the probability of observing the partner’s instantaneous actions into the game-theoretic framework of knowing previous choice outcomes. We find that such “transparency” has a direct influence on the emergence of cooperative behaviours in classic iterated games. The transparent games can contribute to a deeper understanding of social behaviour and decision-making of humans and animals.

## Introduction

One of the most interesting questions in evolutionary biology, social sciences, and economics is the emergence and maintenance of cooperation [1–5]. A popular framework for studying cooperation (or the lack thereof) is game theory, which is frequently used to model interactions between “rational” decision-makers [6–9]. A model for repeated interactions is provided by iterated games with two commonly used settings [2]. In *simultaneous* games all players act at the same time and each player has to make a decision under uncertainty regarding the current choice of the partner(s). In *sequential* games players act one after another in a random or predefined order [10] and the player acting later in the sequence is guaranteed to see the choices of the preceding player(s). Maximal uncertainty only applies to the first player and—if there are more than two players—is reduced with every turn in the sequence.

Both classical settings simplify and restrict the decision context: either no player has any information about the choices of the partners (simultaneous game), or each time some players have more information than others (sequential game). This simplification prevents modelling of certain common behaviours, since humans and animals usually act neither strictly simultaneously nor sequentially, but observe the choices of each other and adjust their actions accordingly [1]. Indeed, the visibility of the partner’s actions plays a crucial role in social interactions, both in laboratory experiments [3, 11–16] and in natural environments [4, 17–20].

**Fig 1:**
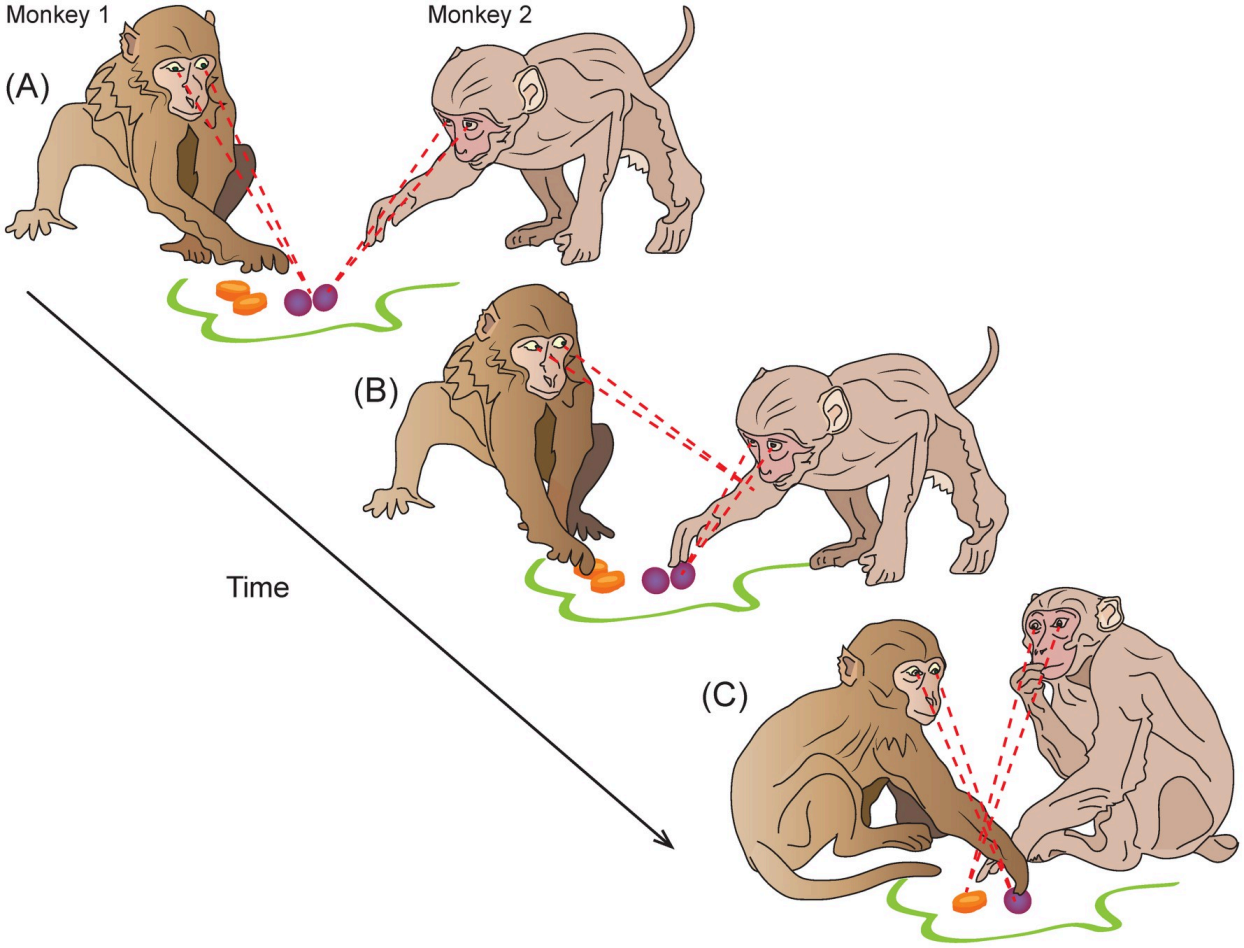
Naturalistic example of a transparent two-player game: Group foraging in monkeys. Two monkeys are reaching for food in two locations that are at some distance so that each monkey can take only one portion. At one location are grapes (preferred food), at the other—a carrot (non-preferred food). (A) Initially both monkeys move toward grapes. (B) Monkey 1 observes Monkey 2 actions and decides to go for the carrot to avoid a potential fight. (C) Next time Monkey 1 moves faster towards the grapes, so Monkey 2 swerves towards the carrot. Coordinated behaviour in such situations has the benefit of higher efficiency and avoids conflicts. This example shows that transparent game is a versatile framework that can be used for describing decision making in social contexts.

For example, in soccer the penalty kicker must decide where to place the ball and the goalkeeper must decide whether to jump to one of the sides or to stay in the centre. Both players resort to statistics about the other’s choices in the past, making this more than a simple one-shot game. Since the goalkeeper must make the choice while the opponent is preparing the shot, a simultaneous game provides a first rough model for such interactions [21, 22]. However, the simultaneous model ignores the fact that both players observe each other’s behaviour and try to predict the direction of the kick or of the goalkeeper’s jump from subtle preparatory cues [15], which often works better than at chance level [21–23]. Using instantaneous cues should not only affect one-shot decisions but also iterative statistics: Learning by observing a keeper over iterations that he has the tendency of jumping prematurely encourages strategies of delayed shots by the kicker, and vice versa. While the soccer example represents a zero-sum game, similar considerations apply to a wide range of real life interactions, see for instance Fig 1. Yet a framework for the treatment of such cases is missing in classical game theory.

To better predict and explain the outcomes of interactions between agents by taking the visibility factor into account, we introduce the concept of transparent games, where players can observe actions of each other. In contrast to the classic simultaneous and sequential games, a *transparent game* is a game-theoretic setting where the access to the information about current choices of other players is probabilistic. For example, for a two-player game in each round three cases are possible:

1. Player 1 knows the choice of Player 2 before making its own choice.
2. Player 2 knows the choice of Player 1 before making its own choice.
3. Neither player knows the choice of the partner.

Only one of the cases 1-3 takes place in each round, but for a large number of rounds one can infer the probability $p_{\text{see}}^{i}$ of Player *i* to see the choice of the partner before making own choice. These probabilities depend on the reaction times of the players. If they act nearly at the same time, neither is able to use the information about partner’s action; but a player who waits before making the choice has a higher probability of seeing the choice of the partner. Yet, explicit or implicit time constraint prevents players from waiting indefinitely for the partner’s choice. In the general case transparent games impose an additional uncertainty on the players acting first: they cannot know in advance whether the other players will see their decision or not in a given round.

The framework of transparent games is generic and includes classic game-theoretical settings as special cases: simultaneous games correspond to $p_{\text{see}}^{1}=p_{\text{see}}^{2}=0$, while sequential games result in $p_{\text{see}}^{1}=0$, $p_{\text{see}}^{2}=1$ for a fixed order of decisions in each round (Player 1 always moves first, Player 2—second) and in $p_{\text{see}}^{1}=p_{\text{see}}^{2}=0.5$ for a random sequence of decisions. Here we ask if probabilistic access to the information on the partner’s choice in transparent games leads to the emergence of different behavioural strategies compared to the fully unidirectional access in sequential games or to the case of no access in simultaneous games.

To answer this question, we consider the effects of transparency on emergence of cooperation in two-player two-choice games. To draw a comparison with the results for classic simultaneous and sequential settings, we focus here on the typically studied memory-one strategies [9, 24] that take into account own and partner’s choices at the previous round of the game. Since cooperation has multiple facets [1, 4, 8], we investigate two types of games which are traditionally used for studying two different forms of cooperation [6, 8, 25, 26]: the iterated Prisoner’s dilemma (iPD) [6] and the iterated (Anti-)Coordination Game (i(A)CG). We chose the generic term (Anti-) Coordination Game because depending on the exact formulation of the payoff matrix, (A)CGs can encompass a wide range of games such as the Battle of the Sexes or Bach-or-Stravinsky game we focus on here [27, 28], but also the Hawk-Dove or Chicken game, and the Leader game [29]. The two games encourage two distinct types of cooperative behaviour [30, 31], since the competitive setting in iPD requires “trust” between partners for cooperation to emerge, i.e. a social concept with an inherent longer-term perspective. In the less competitive i(A)CG, instead, cooperation of players in form of simple coordination of their actions can be beneficial even in one-shot situations. Our hypothesis is that transparency should have differential effects on long-term optimal strategies in these two types of games. We show with the help of evolutionary simulations that this is indeed the case: transparency enhances cooperation in the generally cooperative i(A)CG, but reduces cooperation in the more competitive iPD.

## Results

**Fig 2:**
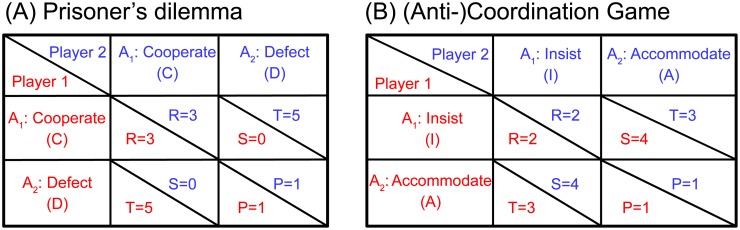
Payoff matrices for Prisoner’s Dilemma and (Anti-)Coordination Game. (A) In Prisoner’s Dilemma, players adopt roles of prisoners suspected of committing a crime and kept in isolated rooms. Due to lack of evidence, prosecutors offer each prisoner an option to minimize the punishment by making a confession. A prisoner can select one of the two actions (*A*_1_ or *A*_2_): either betray the other by defecting (D), or cooperate (C) with the partner by remaining silent. The maximal charge is five years in prison, and the payoff matrix represents the number of years deducted from it (for instance, if both players cooperate (CC, upper left), each gets a two-year sentence, because three years of prison time have been deducted). The letters *R*,*T*,*S* and *P* denote payoff values and stand for Reward, Temptation, Saint and Punishment, respectively. (B) In the (Anti-)Coordination Game variant known as Bach-or-Stravinsky and as Hero [27–29]) two people are choosing between Bach and Stravinsky music concerts. Player 1 prefers Bach, Player 2—Stravinsky, hence, there is an inherent conflict about which concert to choose; yet, above all both prefer going to the concert together. Thus the aim of the players is to *coordinate* (either on Bach or on Stravinsky), which assures maximal joint reward for the players. Players can either insist (I) on their own preference or accommodate (A) the preference of the partner. In these terms, the outcome coordination (attending the same concert) is achieved by selecting complementary actions: either (I, A) or (A, I), which justifies the name: “anti-”coordination. For example, when both agents coordinate on Bach, Player 1 insists, while Player 2 accommodates (I, A). In the “Methods”, we consider also a more general class of (anti-)coordination games, encompassing Hawk-Dove (or Chicken) and Leader.

We investigated the success of different behavioural strategies in the iPD and i(A)CG games by using evolutionary simulations. These simulations allow evaluating long-term optimal strategies using principles of natural selection, where fitness of an individual is defined as the achieved payoff compared to the population average (see “Methods”). The payoff matrices, specifying each player’s payoff conditional upon own and other’s choice, are shown in Fig 2 for both games.

**Fig 3:**
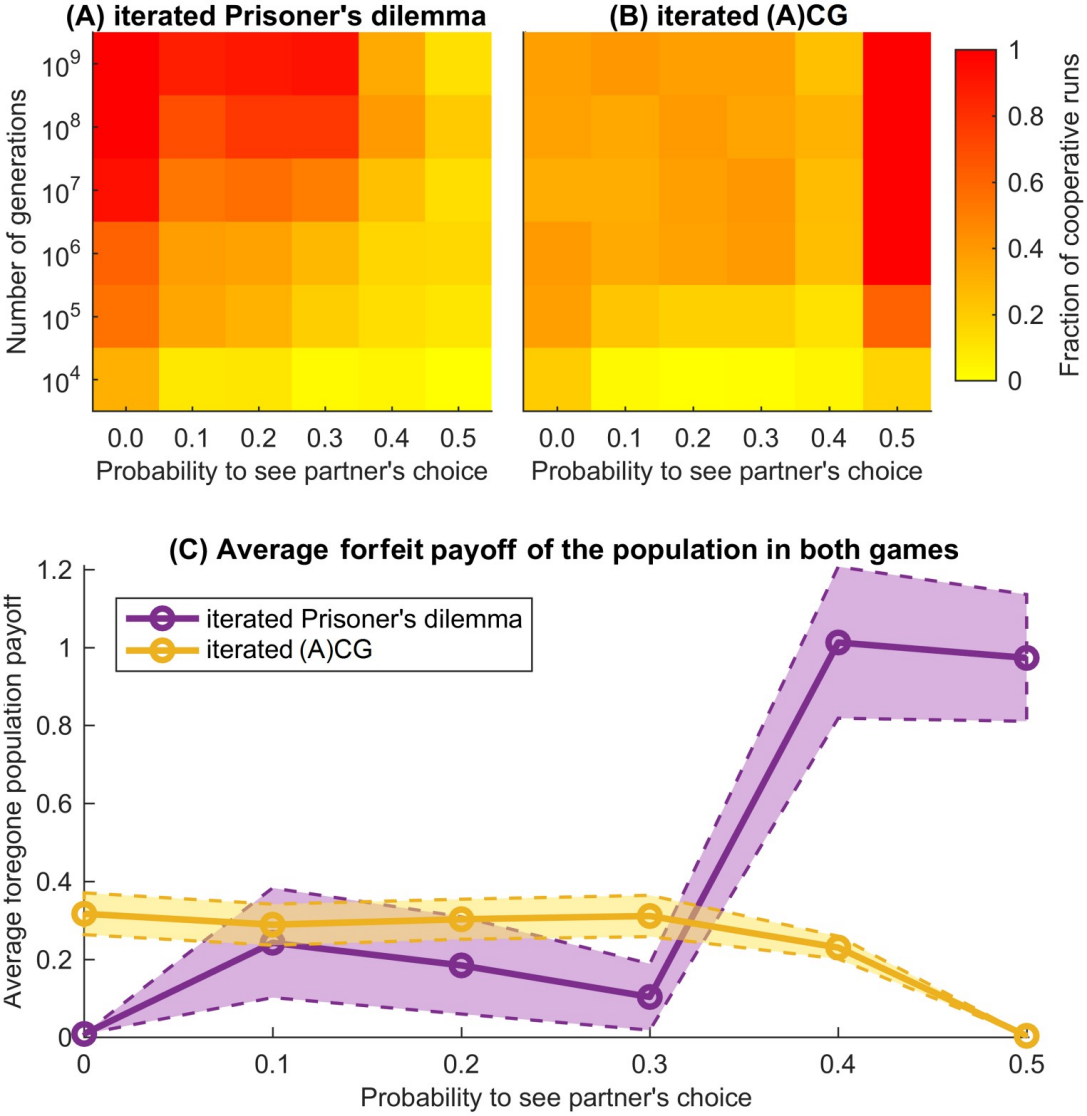
Frequency of establishing effective cooperation and the forfeit reward in the iterated Prisoner’s Dilemma (iPD) and in the iterated (Anti-)Coordination Game (i(A)CG). We performed 80 runs of evolutionary simulations tracing 10^9^ generations of iPD and i(A)CG players. Agents with successful strategies reproduced themselves (had higher fraction in the next generation), while agents with unsuccessful strategies died out, see “Methods” for details. We considered a run as “cooperative” if the average payoff across the population was more than 0.9 times the pay-off of 3 units for cooperative behaviour in iPD [24], and more than 0.95 times the pay-off of 3.5 units for cooperative behaviour in the i(A)CG (i.e., 90% and 95% of the maximally achievable pay-off on average over both players). For i(A)CG we set a higher threshold due to the less competitive nature of this game. (A) In iPD cooperation was quickly established for low probability to see the partner’s choice *p*_see_, but it took longer to develop for moderate *p*_see_ and it drastically decreased for high *p*_see_. (B) In contrast, for i(A)CG frequent cooperation emerges only for high visibility. The small drop in cooperation at *p*_see_ = 0.4 is caused by a transition between two coordination strategies (see main text). (C) The forfeit payoff (maximal possible average payoff of the population minus actual average payoff obtained by the population) further illustrates the same tendencies: higher transparency reduces effectiveness of cooperation in iPD but increases in i(A)CG.

Our evolutionary simulations show that the probability of seeing the partner’s choice had a considerable effect on the likelihood of acting cooperatively. In both games this likelihood differs considerably for the probabilities below and above 0.35 (Fig 3, S2 Note). Further, the transparency levels at which the likelihood of cooperation was high, turned out to be largely complementary in both games.

In the following, we analyse in more detail what is behind the effect of transparency on the cooperation frequency that is revealed in our simulations. First, we provide analytical results for non-iterated (one-shot) transparent versions of Prisoner’s Dilemma (PD) and (Anti-)Coordination Game ((A)CG). Second, after briefly explaining the basic principles adopted in our evolutionary simulations, we describe the strategies that emerge in these simulations for the iPD and i(A)CG games.

### Transparent games without memory: Analytical results

In game theory, the Nash Equilibrium (NE) describes optimal behaviour for the players [7]. In dyadic games, NE is a pair of strategies, such that neither player can get a higher payoff by unilaterally changing its strategy. Both in PD and in (A)CG, players choose between two actions, *A*_1_ or *A*_2_ (see Fig 2): They cooperate or defect in PD and insist or accommodate in (A)CG according to their strategies. In a one-shot transparent game, a strategy is represented by a vector (*s*_1_; *s*_2_; *s*_3_), where *s*_1_ is the probability of selecting *A*_1_ without seeing the partner’s choice, *s*_2_ the probability of selecting A_1_ while seeing the partner also selecting A_1_, and *s*_3_ the probability of selecting A_1_ while seeing partner selecting A_2_, respectively. The probabilities of selecting A_2_ are equal to 1 − *s*_1_, 1 − *s*_2_ and 1 − *s*_3_, correspondingly. For example, strategy (1; 1; 0) in the transparent PD means that the player cooperates unless seeing that the partner defects.

For the one-shot transparent PD we show (Proposition 2 in “Methods”) that all NE are comprised by defecting strategies (0; *x*; 0) with $0\leq x\leq \frac{1-p_{\text{see}}}{p_{\text{see}}}\frac{P-S}{R-S}$, where *P*, *S* and *R* are the elements of the payoff matrix (Fig 2A) and *p*_see_ is the probability to see the choice of the partner. At a population level, this means that cooperation does not survive in the transparent one-shot PD, similar to the classic PD.

For the one-shot transparent (A)CG we show that the NE depend on *p*_see_ (Proposition 4). For $p_{\text{see}}<\frac{T-R}{T+S-2R}$ there are three NE: (a) Player 1 uses (0; 0; 1), Player 2 uses (1; 0; 1); (b) vice versa; (c) both players use strategy (*x*; 0; 1) with $x=\frac{\left(S-P\right)-p_{\text{see}}\left(T+S-2P\right)}{\left(1-2p_{\text{see}}\right)\left(T+S-P-R\right)}$. Note that for the limiting case of *p*_see_ = 0 one gets the three NE known from the classic one-shot simultaneous (A)CG [29]. However, for $p_{\text{see}}\geq \frac{T-R}{T+S-2R}$ the only NE is provided by (1; 0; 1). In particular, for (A)CG defined by the payoff matrix in Fig 2B, there are three NE for *p*_see_ < 1/3 and one NE otherwise. This means that population dynamics is considerably different for the cases *p*_see_ < 1/3 and *p*_see_ > 1/3, and as we show below this is also true for the iterated (A)CG. We also show that transparent versions of two other classical games (“Hawk-Dove” and “Leader”) have a similar NE structure (Proposition 8).

In summary, introducing action transparency influences optimal behaviour already in simple one-shot games.

### Transparent games with memory: Evolutionary simulations

Iterated versions of PD and (A)CG games (iPD and i(A)CG) differ from one-shot games in that the current choice. We focus on strategies taking into account own and partner’s choices in one previous round of the game (“memory-one” strategies) for reasons of tractability. A strategy without memory in transparent games is described by a three-element vector. A memory-one strategy additionally conditions the current choice upon the outcome of the previous round of the game. Since there are four (2 × 2) possible outcomes, a memory-one strategy is represented by a vector $s={\left(s_{k}\right)}_{k=1}^{12}$, where *k* enumerates the twelve (4 × 3) different combinations of previous outcome and the current probability of choice. The entries *s*_*k*_ of the strategy thus represent the conditional probabilities to select action A_1_, specifically

*s*_1_, …, *s*_4_ are probabilities to select A_1_ without seeing the partner’s choice, given that in the previous round the joint choice of the player and the partner was A_1_A_1_, A_1_A_2_, A_2_A_1_, and A_2_A_2_ respectively (the first action specifies the choice of the player, and the second—the choice of the partner);
*s*_5_, …, *s*_8_ are probabilities to select A_1_, seeing the partner selecting A_1_ and given the outcome of the previous round (as before).
*s*_9_, …, *s*_12_ are probabilities to select A_1_, seeing the partner selecting A_2_ and given the outcome of the previous round.

Probabilities to select A_2_ are given by (1 − *s*_*k*_), respectively.

We used evolutionary simulations to investigate which strategies evolve in the transparent iPD and i(A)CG (see “Methods” and [9, 24] for more detail), since an analytical approach would require solving systems of 12 differential equations. We studied an infinite population of players to avoid stochastic effects associated with finite populations [32]. For any generation *t* the population consisted of *n*(*t*) types of players, each defined by a strategy **s**^*i*^ and relative frequency *x*_*i*_(*t*) in the population with $\sum_{i=1}^{n(t)}x_{i}\left(t\right)=1$. To account for possible errors in choices and to ensure numerical stability of the simulations (see “Methods”), we assumed that no pure strategy is possible, that is $\varepsilon \leq s_{k}^{i}\leq 1-\varepsilon$, with *ε* = 0.001 [9, 24]. Frequency *x*_*i*_(*t*) in the population increased with *t* for strategies getting higher-than-average payoff when playing against the current population and decreased otherwise. This ensured “survival of the fittest” strategies. In both games, we assumed players to have equal mean reaction times (see “Methods” for the justification of this assumption). Then the probability *p*_see_ to see the choice of the partner was equal for all players, which in a dyadic game resulted in *p*_see_ ≤ 0.5. We performed evolutionary simulations for various transparencies with *p*_see_ = 0.0, 0.1, …, 0.5.

In the two following sections we discuss the simulation results for both games in detail and describe the strategies that are successful for different transparency levels. Since the strategies in the evolutionary simulations were generated randomly (mimicking random mutations), convergence of the population onto the theoretical optimum may take many generations and observed successful strategies may deviate from the optimum. Therefore, when reporting the results below we employ a coarse-grained description of strategies using the following notation: symbol 0 for *s*_*k*_ ≤ 0.1, symbol 1 for *s*_*k*_ ≥ 0.9, symbol * is used as a wildcard character to denote an arbitrary probability.

To exemplify this notation, let us describe the strategies that are known from the canonical simultaneous iPD [9], affecting exclusively *s*_1_, …, *s*_4_, for the transparent version of this game, i.e. including *s*_5_, …, *s*_12_.

1. The Generous tit-for-tat (**GTFT**) strategy is encoded by (1*a*1*c*;1***;****), where 0.1 < *a*, *c* < 0.9. Indeed, GTFT is characterized by two properties [9]: it cooperates with cooperators and forgives defectors. To satisfy the first property, the probability to cooperate after the partner cooperated in the previous round should be high, thus the corresponding entries of the strategy *s*_1_, *s*_3_, *s*_5_ are encoded by 1. To satisfy the second property, the probability to cooperate after the partner defected should be between 0 and 1. We allow a broad range of values for *s*_2_ and *s*_4_, namely 0.1 ≤ *s*_2_, *s*_4_ ≤ 0.9. We accept arbitrary values for *s*_6_, …, *s*_12_ since for low values of *p*_see_ these entries have little influence on the strategy performance, meaning that their evolution towards optimal values may take especially long. For instance, the strategy entry *s*_7_ is used only when the player has defected in previous round and is seeing that the partner is cooperating in the current round. But GTFT player defects very rarely, hence the *s*_7_ is almost never used and its value has little or no effect on the overall behaviour of a GTFT player.
2. Similarly, Firm-but-fair (**FbF**) by (101*c*;1***;****), where 0.1 < *c* < 0.9.
3. Tit-for-tat (**TFT**) is a “non-forgiving” version of GTFT, encoded by (1010;1***;****).
4. Win–stay, lose–shift (**WSLS**) is encoded by (100*c*;1***;****) with *c* ≥ 2/3. Indeed, in the canonical simultaneous iPD WSLS repeats its own previous action if it resulted in relatively high rewards of *R* = 3 (cooperates after successful cooperation, thus *s*_1_ ≥ 0.9) or *T* = 5 (defects after successful defection, *s*_3_ ≤ 0.1), and switches to another action otherwise (*s*_2_ ≤ 0.1, *s*_4_ = *c* ≥ 2/3). Note that the condition for *s*_4_ is relaxed compared to *s*_2_ since payoff *P* = 1 corresponding to mutual defection is not so bad compared to *S* = 0 and may not require immediate switching. Additionally, we set *s*_5_ ≥ 0.9 to ensure that WSLS players cooperate with each other in the transparent iPD as they do in the simultaneous iPD.
  We also consider a relaxed (cooperative) version of WSLS, which we term “generous WSLS” (**GWSLS**). It follows WSLS principle only in a general sense and is encoded by (1*abc*;1***;****) with *c* ≥ 2/3, *a*, *b* < 2/3 and either *a* > 0.1 or *b* > 0.1.
5. The Always Defect strategy (**AllD**) is encoded by (0000;**00;**00), meaning that the probability to cooperate when not seeing partner’s choice or after defecting is below 0.1, and other behaviour is not specified.

Note that here we selected the coarse-grained descriptions of the strategies, covering only those strategy variants that actually persisted in the population for our simulations.

### Transparency suppresses cooperation in Prisoner’s Dilemma

Results of our simulations for the transparent iPD are presented in Table 1. Most of the effective strategies are known from earlier studies on non-transparent games [9]. They rely on the outcome of the previous round, not on the immediate information about the other player’s choice. However, for high transparency (*p*_see_ → 0.5) a previously unknown strategy emerged, which exploits the knowledge about the other player’s immediate behaviour. We dub this strategy “Leader-Follower” (**L-F**) since when two L-F players meet for *p*_see_ = 0.5, the player acting first (the Leader) defects, while the second player (the Follower) sees this and makes a “self-sacrificing” decision to cooperate. Note that when *mean* reaction times of the players coincide, they have equal probabilities to become a Leader ensuring balanced benefits of exploiting sacrificial second move. We characterized as L-F all strategies with profile (*00*c*;****;*11*d*) with *c* < 1/3 and *d* < 2/3. Indeed, for *p*_see_ = 0.5 these entries are most important to describe the L-F strategy: after unilateral defection the Leader always defects (*s*_2_, *s*_3_ ≤ 0.1) and the Follower always cooperates (*s*_10_, *s*_11_ ≥ 0.9). Meanwhile, mutual defection most likely takes place when playing against a defector, thus both Leaders and Followers have low probability to cooperate after mutual defection (*s*_4_ = *c* < 1/3, *s*_12_ = *d* < 2/3). Behaviour after mutual cooperation is only relevant when a player with L-F is playing against a player with a different strategy, and success of each L-F modification depends on the composition of the population. For instance, (100*c*;111*;100*d*) is optimal in a cooperative population.

**Table 1:** Relative frequencies of strategies that survived for more than 1000 generations in the iterated Prisoner’s Dilemma. The frequencies were computed over 10^9^ generations in 80 runs. The frequency of the most successful strategy for each *p*_see_ value is shown in **bold**.

Strategy		*p*_see_					
name	description	0.0	0.1	0.2	0.3	0.4	0.5
WSLS	(100*c*;1***;****)	**62.9**	**79.8**	**80.3**	**56.3**	12.6	3.8
GWSLS	(1*abc*;1***;****)	0.0	1.0	6.6	22.4	**16.3**	6.0
GTFT	(1*a*1*c*;1***;****)	36.5	0.1	0.1	0.3	0.7	0.5
TFT	(1010;1***;****)	0.0	0.0	0.0	1.9	1.6	1.5
FbF	(101*c*;1***;****)	0.0	0.0	0.1	1.0	6.0	1.8
AllD	(0000;**00;**00)	0.1	0.0	0.0	0.0	2.4	1.9
L-F	(*00*c*;****;*11*d*)	0.0	0.2	0.0	0.0	0.6	**17.8**
Rare transient strategies		0.5	18.9	12.9	18.1	59.8	66.7

**Fig 4:**
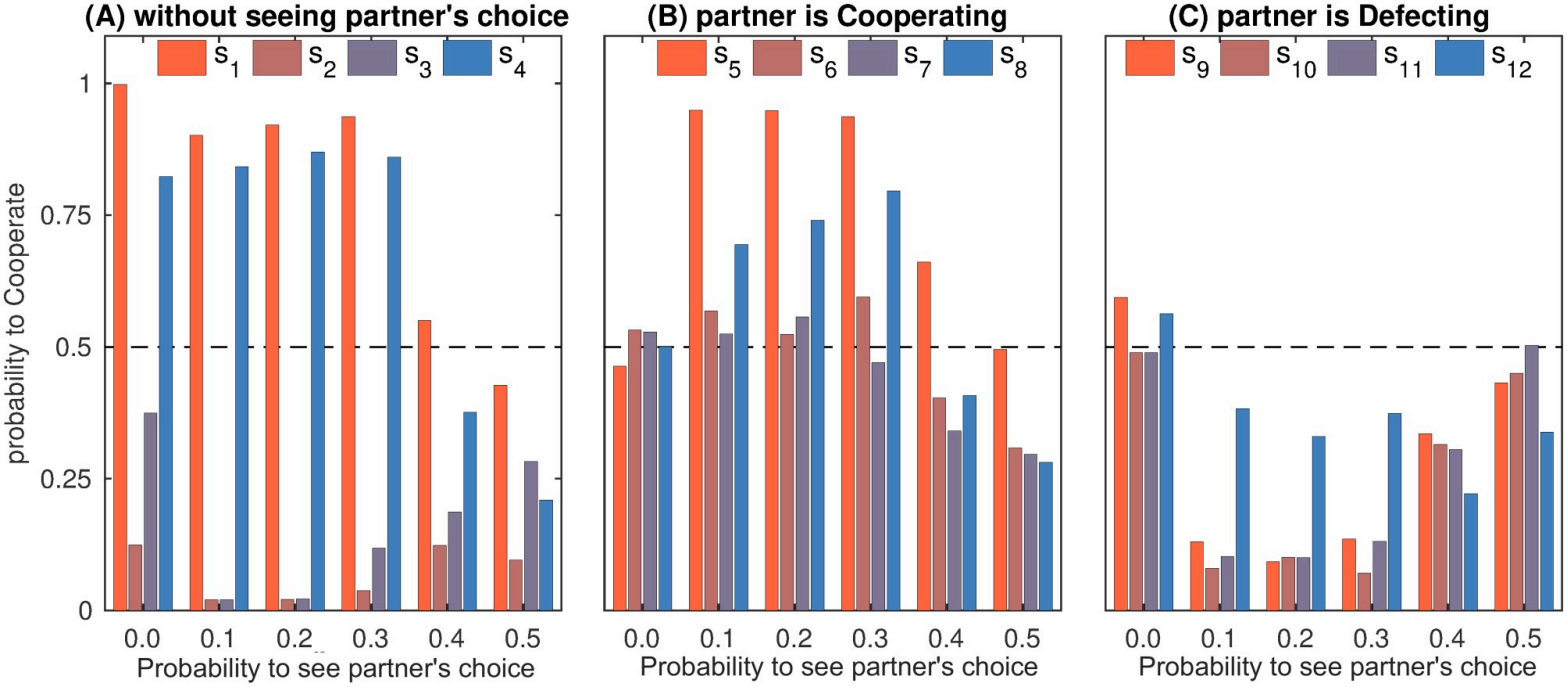
iPD strategies in the final population. Strategies are taken for the 10^9^-th generation and averaged over 80 runs. This figure characterizes the final population as a whole and complements Table 1 representing specific strategies. (A) Strategy entries *s*_1_, …, *s*_4_ are close to (1001) for *p*_see_ = 0.1, …, 0.3 demonstrating the dominance of WSLS. Deviations from this pattern for *p*_see_ = 0.0 and *p*_see_ = 0.4 indicate the presence of the GTFT (1*a*1*b*) and FbF (101*b*) strategies, respectively. For *p*_see_ ≥ 0.4 strategy entries *s*_1_, …, *s*_4_ are quite low due to the extinction of cooperative strategies. (B) Entries *s*_5_, …, *s*_8_ are irrelevant for *p*_see_ = 0.0 (resulting in random values around 0.5) and indicate the same WSLS-like pattern for *p*_see_ = 0.1, …, 0.3. Note that *s*_6_, *s*_7_ > 0 indicate that in transparent settings WSLS-players tend to cooperate seeing that the partner is cooperating even when this is against the WSLS principle. The decrease of reciprocal cooperation for *p*_see_ ≥ 0.4 indicates the decline of WSLS and cooperative strategies in general. (C) Entries *s*_9_, …, *s*_12_ are irrelevant for *p*_see_ = 0.0 (resulting in random values around 0.5) and are quite low for *p*_see_ = 0.1, …, 0.3 (*s*_12_ is irrelevant in a cooperative population). Increase of *s*_9_, …, *s*_11_ for *p*_see_ ≥ 0.4 indicates that mutual cooperation in the population is replaced by unilateral defection.

In summary, as in the simultaneous iPD, WSLS was predominant in the transparent iPD for low and moderate *p*_see_. This is reflected by the distinctive WSLS profiles in the final strategies of the population (Fig 4). Note that GTFT, another successful strategy in the simultaneous iPD, disappeared for *p*_see_ > 0. For *p*_see_ ≥ 0.4, the game resembled the sequential iPD and the results changed accordingly. Similar to the sequential iPD [10, 33, 34], the frequency of WSLS waned, the FbF strategy emerged, cooperation became less frequent and took longer to establish itself (Fig 3A). For *p*_see_ = 0.5 the population was taken over either by L-F, by WSLS-based strategies or (rarely) by FbF or TFT, which is reflected by the mixed profile in Fig 4. Note that the share of distinctly described strategies decreased with increasing *p*_see_, which indicates that for high transparency most strategies appear in the population only transiently and rapidly replace each other, see S1 Fig. Under these circumstances, the relative frequency of L-F (17.8% of the population across all generations) is quite high. The fact that neither L-F nor the transient strategies are generally cooperative explains the drop of cooperation in iPD for high *p*_see_ (Fig 3).

**Fig 5:**
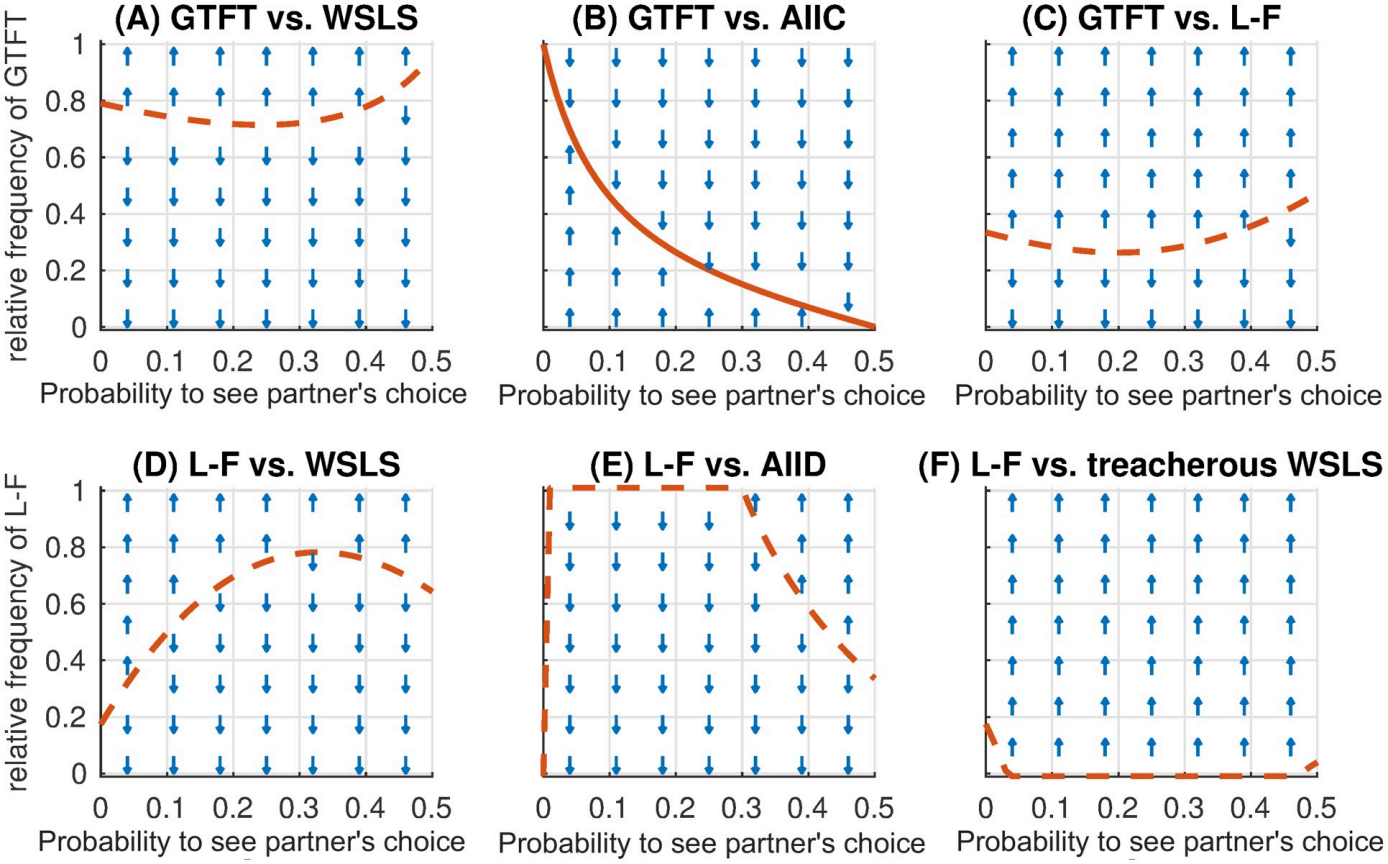
Analytical pairwise comparison of iPD strategies. For each pair of strategies the maps show if the first of the two strategies increases in frequency (up-arrow), or decreases (down-arrow) depending on visibility of the other player’s action and the already existing fraction of the respective strategy. The red lines mark the invasion thresholds, i.e. the minimal fraction of the first strategy necessary for taking over the population against the competitor second strategy. A solid-line invasion threshold shows the stable equilibrium fraction which allows coexistence of both strategies (see “Methods”). Dashed-line invasion thresholds indicate dividing lines above which only the first, below only the second strategy will survive. (A) WSLS $\left(100\frac{9}{10};1001;0000\right)$ has an advantage over GTFT $\left(1\frac{1}{3}1\frac{1}{3};1\frac{1}{3}1\frac{1}{3};0000\right)$: the former takes over the whole population even if its initial fraction is as low as 0.25. (B) GTFT coexists with (prudent) version of cooperative strategy AllC (1111; 1111; 0000), which is more successful for *p*_see_ ≥ 0.1. (C,D) L-F $\left(\frac{1}{3}000;\frac{2}{3}000;111\frac{1}{3}\right)$ performs almost as good as GTFT and WSLS, (E) but can resist the AllD strategy (0000; 0000; 0000) only for high transparency. (F) Note that WSLS may lapse into its treacherous version, $\left(100\frac{9}{10};0000;0000\right)$. This strategy dominates WSLS for *p*_see_ > 0 but is generally weak and cannot invade when other strategies are present in the population. Notably, when treacherous WSLS takes a part of the population, it is quickly replaced by L-F, which partially explains L-F success for high *p*_see_.

To better explain the success of different strategies at different transparency levels, we analytically compared the strategies that emerged most frequently in simulations. Pairwise comparison of these strategies (Fig 5) helps to explain the superiority of WSLS for *p*_see_ < 0.5, the disappearance of GTFT for *p*_see_ > 0.0, and the abrupt increase of L-F frequency for *p*_see_ = 0.5.

**Fig 6:**
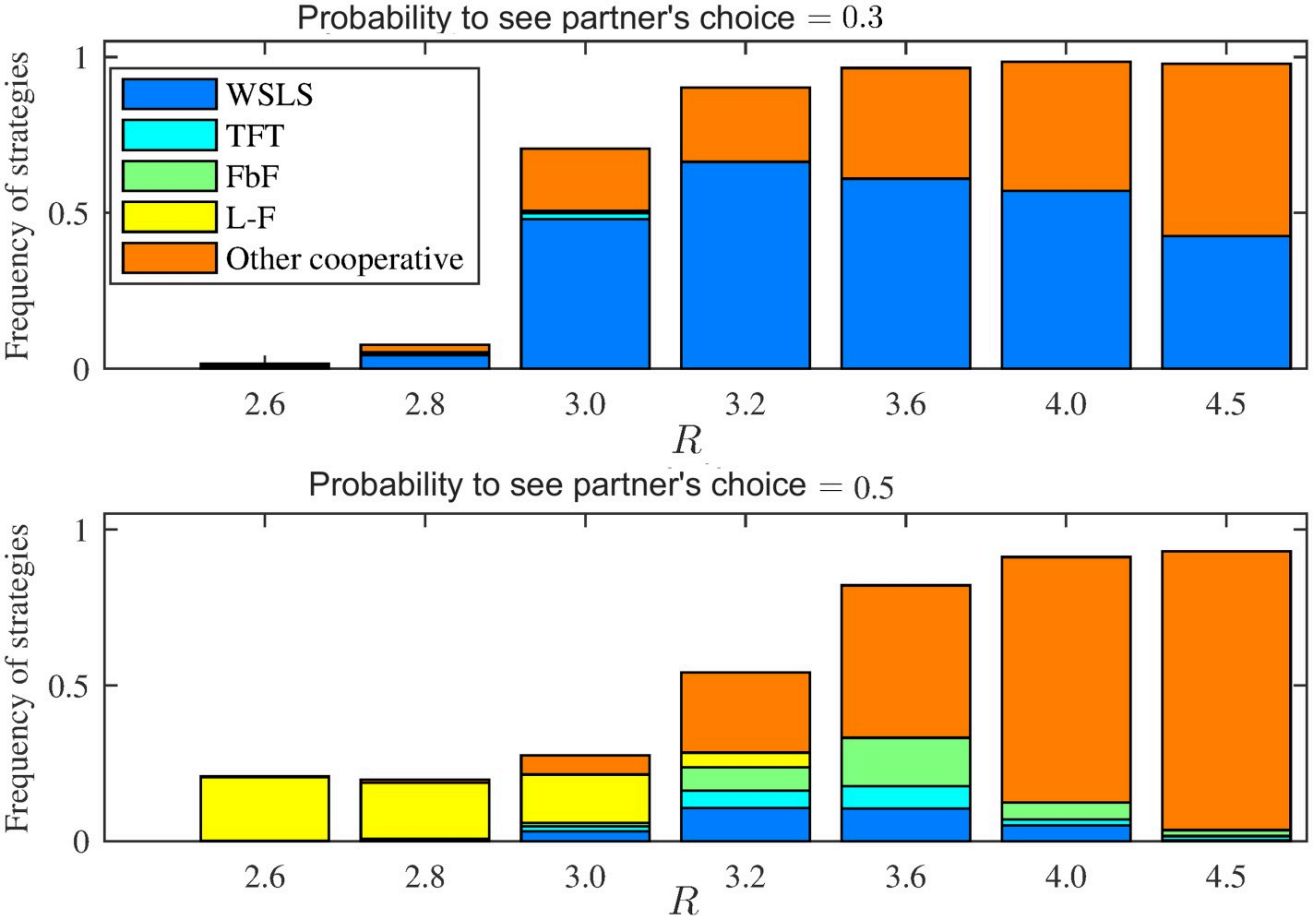
Frequencies of strategies that survived for more than 1000 generations after they emerged in the iterated Prisoner’s Dilemma population as function of reward *R* for mutual cooperation. Data exemplified for *p*_see_ = 0.3 and for *p*_see_ = 0.5. Values of *T*, *S* and *P* are the same as in Fig 2, values of *R* are in range (*S* + *T*)/2 < *R* < *T* that defines the Prisoner’s Dilemma payoff. The frequencies were computed over 10^9^ generations in 40 runs. We describe as “other cooperative” all strategies having a pattern (1*1*;1***;****) or (1**1;1***;****) but different from WSLS, TFT and FbF. While for *p*_see_ = 0.3 population for low *R* mainly consists of defectors, for *p*_see_ = 0.5 L-F provides an alternative to defection. For *R* ≥ 3.2 mutual cooperation becomes much more beneficial, which allows cooperative strategies to prevail for all transparency levels. Yet higher transparency reduces cooperation for all values of *R*. Note that the higher *R* is the less specific the cooperative strategies are. Indeed, for high *R* cooperation is much more effective than other types of behaviour, which makes all cooperative strategies (including even unconditional cooperation) evolutionary successful (we refer to [24] for a similar result in the case of sequential iPD).

Although cooperation in the transparent iPD is rare for *p*_see_ ≥ 0.4, L-F is in a sense also a cooperative strategy for iPD: In a game between two L-F players with equal mean reaction times, both players alternate between unilateral defection and unilateral cooperation in a coordinated way, resulting in equal average payoffs of (*S* + *T*)/2. Such alternation is generally sub-optimal in iPD since *R* > (*S* + *T*)/2; for instance, in our simulations *R* = 3 > (*S* + *T*)/2 = 2.5. To check the influence of the payoff on the strategies predominance, we have varied values of *R* by keeping *T*, *S* and *P* the same as in Fig 2 as it was done in [24] for simultaneous iPD. Fig 6 shows that for *R* > 3.2, evolution in the transparent iPD strongly favours cooperation for all transparency levels, but *R* ≤ 3.2 is sufficiently close to (*S* + *T*)/2 to make L-F a safe and efficient strategy. Indeed, as one can see from Fig 3C for *R* = 3, other strategies for high transparency perform much worse than L-F resulting in average population payoff around 2.

Note that higher transparency reduces cooperation for all values of *R*, although the effect is most prominent for *R* ≤ 3.2. Indeed, while the share of non-cooperative strategies in the population is negligible for *R* ≥ 3.6 with *p*_see_ = 0 [24] and is below 5% with *p*_see_ = 0.3, it is above 5% for *p*_see_ = 0.5 for all *R* ≥ 3.6 (compare the top and bottom plots in Fig 6).

### Cooperation emergence in the transparent (Anti-)Coordination Game

Our simulations revealed that four memory-one strategies are most effective in i(A)CG for various levels of transparency. In contrast to iPD there exist only few studies of strategies in non-transparent i(A)CG [31, 35], therefore we describe the observed strategies in detail.

1. **Turn-taker** aims to enter a fair coordination regime, where players alternate between IA (Player 1 insists and Player 2 accommodates) and AI (Player 1 accommodates and Player 2 insists) states. In the simultaneous i(A)CG, this strategy takes the form (*q*01*q*), where *q* = 5/8 guarantees maximal reward in a non-coordinated play against a partner with the same strategy for the payoff matrix in Fig 2B. Turn-taking was shown to be successful in the simultaneous i(A)CG for a finite population of agents with pure strategies (i.e., having 0 or 1 entries only, with no account for mistakes) and a memory spanning three previous rounds [31]. Here in our transparent i(A)CG, we classify as Turn-takers all strategies encoded by (*01*;*0**;**1*).
2. Challenger takes the form (1101) in the simultaneous i(A)CG. When two players with this strategy meet, they initiate a “challenge”: both insist until one of the players makes a mistake (that is, accommodates). Then, the player making the mistake (loser) submits and continues accommodating, while the winner continues insisting. This period of unfair coordination beneficial for the winner ends when the next mistake of either player (the winner accommodating or the loser insisting) triggers a new “challenge”. Challenging strategies were theoretically predicted to be successful in simultaneous i(A)CG [35, 36]. In our transparent i(A)CG, the challenger strategy is encoded by (11b*;****;*1**) and has two variants: **Challenger** “obeys the rules” and does not initiate a challenge after losing (*b* ≤ 0.1), while **Aggressive Challenger** may switch to insisting even after losing (0.1 < *b* ≤ 1/3). In both variants we allow a broad range of values for $s_{4}^{i}$ since this entry is used after both players accommodate, which is an extremely rare case in a game between challengers.
3. The Leader-Follower (**L-F**) strategy **s** = (1111; 0000; 1111) relies on the visibility of the other’s action and was not considered previously. In the i(A)CG game between two players with this strategy, the faster player insists and the slower player accommodates. In a simultaneous setting, this strategy lapses into inefficient stubborn insisting since all players consider themselves leaders, but in transparent settings with high *p*_see_ this strategy provides an effective and fair cooperation if *mean* reaction times are equal. In particular, for *p*_see_ > 1/3 the L-F strategy is a Nash Equilibrium in a one-shot game (see Proposition 4 in “Methods”).
  When the entire population adopts an L-F strategy, most strategy entries become irrelevant since in a game between two L-F players the faster player never accommodates and the outcome of the previous round is either IA or AI. Therefore, we classify all strategies encoded by (*11*;*00*;****) as L-F.
4. **Challenging Leader-Follower** is a hybrid of the Challenger and L-F strategies encoded by (11b*;0*c*0*;*1**), where 1/3 < *b* ≤ 0.9, *c* ≤ 1/3. With such a strategy a player tends to insist without seeing the partner’s choice, and tends to accommodate when seeing that the partner insists; both these tendencies are stronger than for Aggressive Challengers, but not as strong as for Leader-Followers.

**Fig 7:**
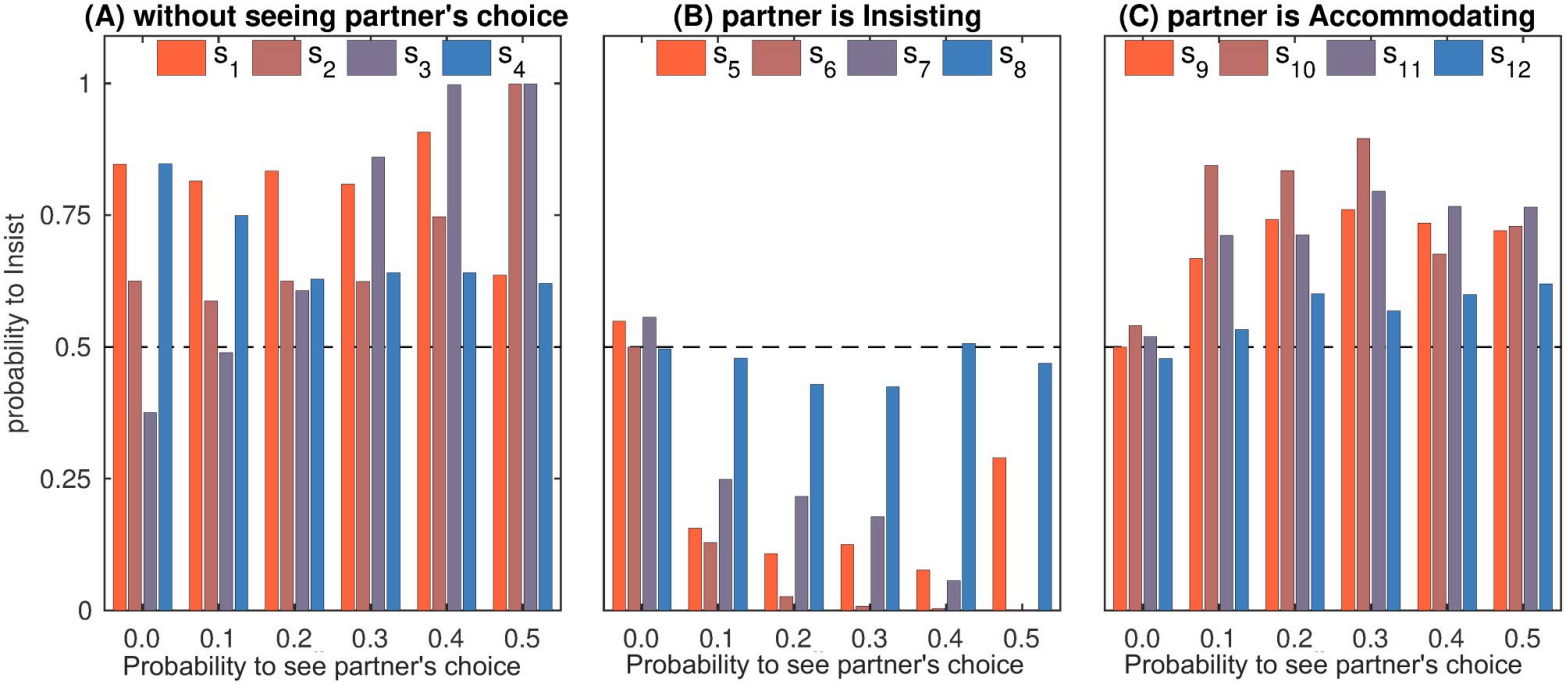
i(A)CG strategies in the final population. Strategies are taken for the 10^9^-th generation and averaged over 80 runs. This figure characterizes the final population as a whole and complements Table 2 representing specific strategies. (A): Strategy entries *s*_1_, …, *s*_4_. The decrease of the *s*_2_/*s*_3_ ratio reflects the transition of the dominant strategy from challenging to turn-taking for *p*_see_ = 0.0, …, 0.4. For *p*_see_ = 0.5 the dominance of the Leader-Follower strategy is indicated by *s*_2_ = *s*_3_ = 1. (B) Entries *s*_5_, …, *s*_8_ are irrelevant for *p*_see_ = 0. Values of *s*_6_, *s*_7_ decrease as *p*_see_ increases, indicating an enhancement of cooperation in i(A)CG for higher transparency (*s*_8_ is almost irrelevant since mutual accommodation is a very rate event, and *s*_5_ is irrelevant for a population of L-F players taking place for *p*_see_ = 0.5). (C) Entries *s*_9_, …, *s*_12_ are irrelevant for *p*_see_ = 0. The decrease of the *s*_10_/*s*_11_ ratio for *p*_see_ = 0.1, …, 0.4 reflects the transition of the dominant strategy from challenging to turn-taking.

The results of our simulations for i(A)CG are presented in Table 2. The entries of the final population average strategy (Fig 7) show considerably different profiles for various values of *p*_see_. Challengers, Turn-takers, and Leader-Followers succeeded for low, medium and high probabilities to see partner’s choice, respectively. Note that due to the emergence of Leader-Follower strategy, cooperation thrives for *p*_see_ = 0.5 and is established much faster than for lower transparency (Fig 3B).

**Table 2:** Relative frequencies of strategies that survived for more than 1000 generations in the (Anti-)Coordination Game. The frequencies were computed over 10^9^ generations in 80 runs. The frequency of the most successful strategy for each *p*_see_ value is shown in **bold**.

Strategy		*p*_see_					
name	description	0.0	0.1	0.2	0.3	0.4	0.5
Turn-taker	(*01*;*0**;**1*)	37.5	41.2	**37.5**	**37.5**	24.9	0.0
Challenger	(11b*;****;*1**)	**62.5**	**42.7**	3.4	0.5	0.0	0.0
Aggressive Challenger	(11b*;****;*1**)	0.0	14.4	30.7	3.3	0.0	0.0
Challenging Leader-Follower	(11b*;0*c*0*;*1**)	0.0	1.1	25.2	31.8	0.0	0.0
Leader-Follower	(*11*;*00*;****)	0.0	0.1	2.5	26.1	**74.6**	**100.0**
Rare transient strategies		0.0	0.5	0.7	0.8	0.5	0.0

**Fig 8:**
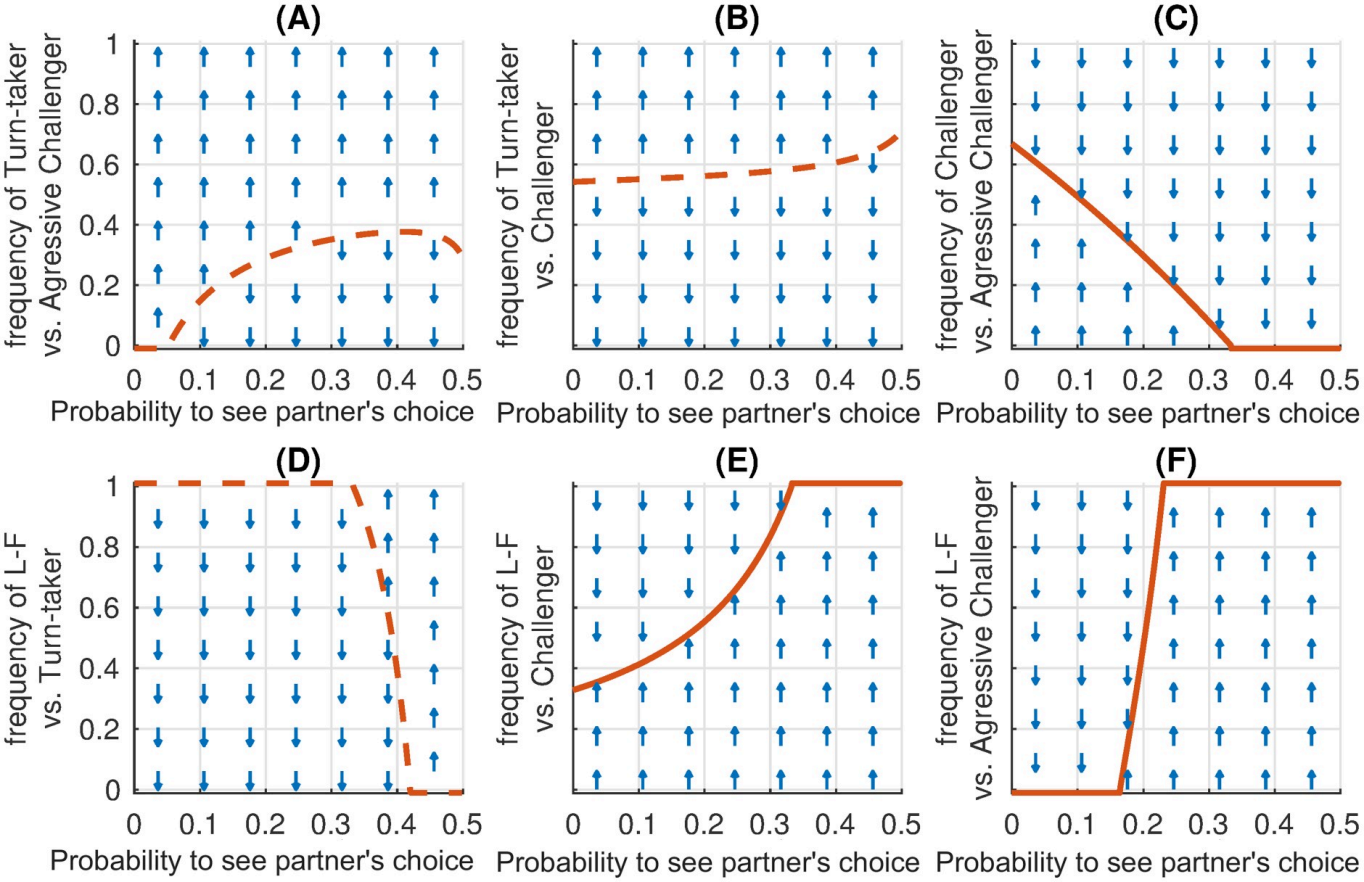
Analytical pairwise comparison of i(A)CG strategies. For each pair of strategies the maps show if the first of the two strategies increases in frequency (up-arrow), or decreases (down-arrow) depending on visibility of the other player’s action and the already existing fraction of the respective strategy. The red lines mark the invasion thresholds, i.e. the minimal fraction of the first strategy necessary for taking over the population against the competitor second strategy. Solid-line invasion thresholds show the stable equilibrium fraction which allows coexistence of both strategies (see “Methods”). Dashed-line invasion thresholds indicate dividing lines above which only the first, below only the second strategy will survive. In all strategies, 1 stands for 0.999 and 0—for 0.001, the entries *s*_9_ = … = *s*_12_ = 1 are the same for all strategies and are omitted. (A) Turn-taker (*q*01*q*; 0000) with *q* = 5/8 for *p*_see_ > 0 outperforms Aggressive Challenger $\left(11\frac{1}{5}1;\frac{1}{2}\frac{1}{2}\frac{1}{5}\frac{1}{2}\right)$, (B) but not Challenger $\left(\frac{9}{10}101;\frac{1}{2}\frac{1}{2}0\frac{1}{2}\right)$. (C) Challenger can coexist with Aggressive Challenger for low transparency, but is dominated for *p*_see_ > 1/3. (D) Leader-Follower (1111; 0000) clearly outperforms Turn-taker for *p*_see_ > 0.4 and (E,F) other strategies for *p*_see_ > 1/3.

To provide additional insight into the results of the i(A)CG simulations, we studied analytically how various strategies perform against each other (Fig 8). As with the iPD, this analysis helps to understand why different strategies were successful at different transparency levels. A change of behaviour for *p*_see_ > 1/3 is in line with our theoretical results (Corollary 7) indicating that for these transparency levels L-F is a Nash Equilibrium. Population dynamics for i(A)CG with a payoff different from the presented in Fig 2B also depends on the Nash Equilibria of one-shot game, described by Proposition 4 in “Methods”.

## Discussion

In this paper, we introduced the concept of transparent games which integrates the visibility of the partner’s actions into a game-theoretic setting. As a model case for transparent games, we considered iterated dyadic games where players have probabilistic access to information about the partner’s choice in the current round. When reaction times for both players are equal on average, the probability *p*_see_ of accessing this information can vary from *p*_see_ = 0.0 corresponding to the canonical simultaneous games, to *p*_see_ = 0.5 corresponding to sequential games with random order of choices. Note that in studies on the classic sequential games [10, 33] players were bound to the same strategy regardless of whether they made their choice before or after the partner. In contrast, transparent games allow different sub-strategies (*s*_1_, …, *s*_4_), (*s*_5_, …, *s*_8_) and (*s*_9_, …, *s*_12_) for these situations.

We showed that even a small probability *p*_see_ of seeing the partner’s choice before one’s own decision changes the long-term optimal behaviour in the iterated Prisoner’s Dilemma (iPD) and (Anti-)Coordination Game (i(A)CG). When this probability is high, its effect is pronounced: transparency enhances cooperation in the generally cooperative i(A)CG, but reduces cooperation in the more competitive iPD. Different transparency levels also bring qualitatively different strategies to success. In particular, in both games for high transparency a new class of strategies, which we termed “Leader-Follower” strategies, evolves. Although frequently observed in humans and animals (see, for instance, [5, 13], these strategies have up to now remained beyond the scope of game-theoretical studies, but naturally emerge in our transparent games framework. Note that here we focused on memory-one strategies for the reasons of better tractability, results for strategies with longer memory can differ considerably [37].

Our approach is similar to the continuous-time approaches suggested in [38] and [39]. However, in these studies the game is played continuously, without any rounds at all, while here we suppose that the game consists of clearly specified rounds, although the time within each round is continuous. This assumption can be considered naturalistic, since many real world interactions and behaviours are episodic, have clear starting and end points, and hence are close to distinct rounds [4, 14, 40, 41]. Transparent games to some degree resemble random games (see e.g. [42, 43]) since in both settings the outcome of the game depends on a stochastic factor. However in random games randomness immediately affects the payoff, while in transparent games it determines the chance to learn the partner’s choice. While this chance influences the payoff of the players, the effect depends on their strategies, which is not the case in random games. Transparent games are also related to Bayesian games (see e.g. [44–47]), where players are uncertain about the rules of the game (payoff, information possessed by other players, etc.) but have subjective probability distributions over the possible alternatives (beliefs). While in Bayesian games players dynamically update their beliefs by learning [45, 48], in this manuscript we consider static agents, and the dynamics happens only on the population level. Yet, introducing learning mechanisms [49] into the framework of transparent games could be an interesting direction for future work. More generally, transparent games can be considered as a special case of games with imperfect or asymmetric information, which have been studied before, mainly in economics (see [50–54] for recent examples). In games with private monitoring, for example (see [55] for a review), each player gets imperfect information on the actions of other players at the end of every round. Different players may get different information, which is similar to our transparent games. Yet, our approach differs from those developed in economic game theory, since in the transparent games players have different information about the immediately relevant *present* actions, while in games with imperfect private monitoring players have different information about the *past* actions [50, 53–55]. This difference is intended and important, since reaction times and direct action visibility are relevant in natural interactive behaviours as studied in biology and neuroscience, while it might be of less importance in economics. However, the relation of transparent games to games with imperfect private monitoring helps to make an interesting observation: The information available to the players in the transparent games is inherently asymmetric in those rounds where choice of one player is visible to the other (although here we consider players as getting the same amount of information on *on average*, across many rounds). Thus, high transparency also means high asymmetry of the access to the information in each specific round. This asymmetry (and not the amount information per se) may be the actual cause of the shift in the optimal behaviour observed for the high transparency.

The value of probability *p*_see_ strongly affects the evolutionary success of strategies. In particular, in the transparent i(A)CG even moderate *p*_see_ helps to establish cooperative turn-taking, while high *p*_see_ brings about a new successful strategy, Leader-Follower (L-F). For the transparent iPD we have shown that for *p*_see_ > 0 the Generous tit-for-tat strategy is unsuccessful and Win–stay, lose–shift (WSLS) is an unquestionable evolutionary winner for 0 < *p*_see_ ≤ 0.4. However, WSLS is not evolutionary stable (see the caption of Fig 5); our results indicate that in general there are no evolutionary stable strategies in the transparent iPD, which was already known to be the case for the simultaneous iPD [9, 56]. Moreover, if reward for mutual cooperation *R* ≤ 3, for high transparencies (*p*_see_ ≥ 0.4) all strategies become quite unstable and cooperation is hard to establish (Fig 6). Finally, for *p*_see_ = 0.5, L-F becomes successful in iPD and is more frequent than WSLS for *R* ≤ 3.2. For such a payoff, mutual cooperation is not much more beneficial than the alternating unilateral defection resulting from the L-F strategy. It brings a payoff of (*S* + *T*)/2 = 2.5, but is generally less susceptible to exploitation by defecting strategies. This explains the abrupt drop of cooperation in the transparent iPD with *p*_see_ ≥ 0.4 for *R* = 3.0 (S2 Note), while such a drop is less prominent for *R* > 3.2 (Fig 6). Note that *R* > 3 > (*T* + *P*)/2 promotes mutual cooperation among memory-one strategies since it results in higher payoff than defecting a cooperator (resulting in a payoff *T*) followed by mutual defection (payoff *P*), which is a natural response of any cautious strategy like TFT or WSLS. Therefore the case *R* > 3.2 is perhaps less interesting than the classic payoff matrix with *R* = 3.

Although resulting in a lower payoff than explicit cooperation, L-F can be also seen as a cooperative strategy for iPD. While the choice of Leaders (defection) is entirely selfish, Followers “self-denyingly” cooperate with them. Importantly, the L-F strategy is not beneficial for some of the players using it in any finite perspective, which distinguishes this strategy from most cooperative strategies. Let us explain this point by comparing L-F with WSLS. In a game between two WSLS-players, neither benefits from unilaterally switching to defection even in a short term for *R* ≥ (*T* + *P*)/2. While the defecting player gets *T* = 5 in the first round, its payoff in the next round is *P* = 1, which makes the average payoff over two rounds less than or equal to the reward for cooperation *R*. Thus for the iPD with standard payoff *R* = 3 = (*T* + *P*)/2 WSLS players do not benefit from defecting their WSLS-partners already for the two-round horizon. (Note that for *R* < (*T* + *P*)/2 defection is effective against WSLS, which explains the low frequency of WSLS for *R* < 3 in Fig 6). Now, assume that one is playing the transparent iPD with *p*_see_ = 0.5 against a partner with a pure L-F strategy (0000; 1111; 1111) and has to choose between L-F and AllD strategies. In a single round using AllD is (strictly) better with probability *p* = 1/2 (probability of being a Follower). From the two-round-perspective using AllD is beneficial with *p* = 1/4 (the probability of being a Follower in both rounds). For *n* = 6 rounds, AllD is still better than L-F with *p* = 7/64 (the probability of being a Follower in 5 or 6 rounds out of 6, which results in an average payoff equal to either 5/6 or 0). In general, for any finite number *n* of rounds, there is a risk to suffer from using the L-F strategy instead of AllD, and the probability of this is given by $\sum_{k=0}^{\left\lceil nP/T\right\rceil }C_{k}^{n}$, where ⌈*nP*/*T*⌉ is the integer part of *nP*/*T* and $C_{k}^{n}=\frac{n!}{k!(n-k)!}$ is a binomial coefficient. That is adhering to L-F is not beneficial for some of the L-F players in any finite horizon, which makes their behaviour in a sense altruistic. Our results for the transparent iPD demonstrate that such “altruistic-like” behaviour may evolve in a population even without immediate reciprocation. The inherently unequal payoff distribution among L-F players for a final number of rounds opens interesting perspectives for research, but is outside the scope of this manuscript.

The lack of stability in the transparent iPD renders the analysis of the strategy dynamics for this game non-trivial. Therefore we do not provide here an exhaustive description of strategies in iPD and content ourselves with general observations and explanations. An in-depth analysis of strategy dynamics in the transparent iPD will be provided elsewhere as a separate, more technical paper [57].

Despite the clear differences between the two games, predominant strategies evolving in iPD and i(A)CG have some striking similarities. First of all, in both games, L-F appears to be the most successful strategy for high *p*_see_ (although for iPD with *R* ≤ 3 the share of Leader-Followers in the population across all generations is only about 20%, other strategies are even less successful as most of them appear just transiently and rapidly replace each other). This prevalence of the L-F strategy can be explained as follows: in a group where the behaviour of each agent is visible to the others and can be correctly interpreted, group actions hinge upon agents initiating these actions. In both games these initiators are selfish, but see S2 Note for an example of an “altruistic” action initiation. For low and moderate values of *p*_see_ the similarities of the two games are less obvious. However, the Challenger strategy in i(A)CG follows the same principle of “Win–stay, lose–shift” as the predominant strategy WSLS in iPD, but with modified definitions of “win” and “lose”. For Challenger winning is associated with any outcome better than the minimal payoff corresponding to the mutual accommodation. Indeed, a Challenger accommodates until mutual accommodation takes place and then switches to insisting. Such behaviour is described as “modest WSLS” in [35, 58] and is in-line with the interpretation of the “Win–stay, lose–shift” principle observed in animals [59].

The third successful principle in the transparent iPD is “Tit-for-tat”, embodied in Generous tit-for-tat (GTFT), TFT and Firm-but-fair (FbF) strategies. This principle also works in both games since turn-taking in i(A)CG is nothing else but giving tit for tat. In particular, the TFT and FbF strategies, which occur frequently in iPD for *p*_see_ ≥ 0.4, are partially based on taking turns and are similar to the Turn-Taker strategy in i(A)CG. The same holds to a lesser extent for the GTFT strategy.

The success of specific strategies for different levels of *p*_see_ makes sense if we understand *p*_see_ as a species’ ability to signal intentions and to interpret these signals when trying to coordinate (or compete). The higher *p*_see_, the better (more probable) is the explicit coordination. This could mean that a high ability to explicitly coordinate actions leads to coordination based on observing the leader’s behaviour. In contrast, moderate coordination ability results in some form of turn-taking, while low ability leads to simple strategies of WSLS-type. In fact, an agent utilizing the WSLS principle does not even need to comprehend the existence of the second player, since WSLS “embodies an almost reflex-like response to the pay-off” [24]. The ability to cooperate may also depend on the circumstances, for example, on the physical visibility of partner’s actions. In a relatively clear situation, following the leader can be the best strategy. Moderate uncertainty requires some (implicit) rules of reciprocity embodied in turn-taking. High uncertainty makes coordination difficult or even impossible, and may result in a seemingly irrational “challenging behaviour” as we have shown for the transparent i(A)CG. However, when players can succeed without coordination (which was the case in iPD), high uncertainty about the other players’ actions does not cause a problem.

While we focused here on the iterated Prisoner’s Dilemma and on the specific formulation of iterative (Anti-)Coordination Game, the transparent games framework can be applied to treatment of other two-player, two-action transparent games. In particular, we have shown that the structure of the Nash equilibria for the Hawk-Dove and Leader games is identical to that of (Anti-)Coordination Game (see Methods, Proposition 8), which suggests that the transparency has a strong effect on successful strategies in these games as well. As a future work, it would also be interesting to extend the transparent game framework to N-agent interactions [60–62], to provide an account of naturalistic dynamics in groups.

By taking the visibility of the agents’ actions into account, transparent games may offer a compelling theoretical explanation for a range of biological, sociological and psychological phenomena. One potential application of transparent games is related to experimental research on social interactions, including the emerging field of social neuroscience that seeks to uncover the neural basis of social signalling and decision-making using neuroimaging and electrophysiology in humans and animals [63–66]. So far, most studies have focused on sequential [67, 68] or simultaneous games [69]. One of the main challenges in this field is extending these studies to direct real-time interactions that would entail a broad spectrum of dynamic competitive and cooperative behaviours. In line with this, several recent studies also considered direct social interactions in humans and non-human primates [12–14, 41, 70–74] during dyadic games where players can monitor actions and outcomes of each other. Transparent games allow modelling the players’ access to social cues, which is essential for the analysis of experimental data in the studies of this kind [8]. This might be especially useful when behaviour is explicitly compared between “simultaneous” and “transparent” game settings, as in [12, 14, 70, 74]. In particular, the enhanced cooperation in the transparent i(A)CG for high *p*_see_ provides a theoretical explanation for the empirical observations in [14], where humans playing an i(A)CG-type game demonstrated a higher level of cooperation and a fairer payoff distribution when they were able to observe the actions of the partner while making their own choice. In view of the argument that true cooperation should benefit from enhanced communication [8], the transparent i(A)CG can in certain cases be a more suitable model for studying cooperation than the iPD (see also [75, 76] for a discussion of studying cooperation by means of i(A)CG-type games).

In summary, transparent games provide a theoretically attractive link between classical concepts of simultaneous and sequential games, as well as a computational tool for modelling real-world interactions. This approach allows integrating work on sensorimotor decision-making under uncertainty with economic game theory. We thus expect that the transparent games framework can help to establish a deeper understanding of social behaviour in humans and animals.

## Methods

### Transparent games between two players

In this study, we focus on iterated two-player (dyadic) two-action games: in every round both players choose one of two possible actions and get a payoff depending on the mutual choice according to the payoff matrix (Fig 2). A new game setting, *transparent game*, is defined by a payoff matrix and probabilities $p_{\text{see}}^{i}$ (*i* = 1, 2) of Player *i* to see the choice of the other player, $0\leq p_{\text{see}}^{1},p_{\text{see}}^{2}\leq 1$. Note that $p_{\text{see}}^{1}+p_{\text{see}}^{2}\leq 1$, and $\left(1-p_{\text{see}}^{1}-p_{\text{see}}^{2}\right)$ is the probability that neither of players knows the choice of the partner because they act sufficiently close in time so that neither players can infer the other’s action prior to making their own choice. The probabilities $p_{\text{see}}^{i}$ can be computed from the distributions of reaction times for the two players, as shown in S1 Note for reaction times modelled by exponentially modified Gaussian distribution [77, 78]. In this figure, reaction times for both players have the same mean, which results in symmetric distribution of reaction time differences (SN1 Fig. 1B in S1 Note) and $p_{\text{see}}^{1}=p_{\text{see}}^{2}\leq 0.5$. Here we focus only on this case since for both games considered in this study, unequal mean reaction times provide a strong advantage to one of the players (see below). However, in general $p_{\text{see}}^{1}\neq p_{\text{see}}^{2}$.

To illustrate how transparent, simultaneous and sequential games differ, let us consider three scenarios for a Prisoner’s Dilemma (PD):

1. If prisoners write their statements and put them into envelopes, this case is described by simultaneous PD.
2. If prisoners are questioned in the same room in a random or pre-defined order, one after another, this case is described by sequential PD.
3. Finally, in a case of a face-to-face interrogation where prisoners are allowed to answer the questions of prosecutors in any order (or even to talk simultaneously) the transparent PD comes into play. Here prisoners are able to monitor each other and interpret inclinations of the partner in order to adjust their own choice accordingly.

While the transparent setting can be used both in zero-sum and non-zero-sum games, here we concentrate on the latter class where players can cooperate to increase their joint payoff. We consider the transparent versions of two classic games, the PD and the (Anti-)Coordination Game ((A)CG). We have selected PD and (A)CG as representatives of two distinct types of symmetric non-zero-sum games [30, 31]: maximal joint payoff is awarded when players select the **same** action (cooperate) in PD, but **complementary** actions in (A)CG (one insists on own preferred option, and the other accommodates this option, to achieve common goal). In games of (A)CG type, one of the two coordinated choices is more beneficial for Player 1 (Player 1 insists, Player 2 accommodates), and the other for Player 2 (Player 1 accommodates, Player 2 insists), thus to achieve fair cooperation players should alternate between these two states.

Another important difference between the two considered games is that in (A)CG a player benefits from acting before the partner, while in PD it is mostly preferable for a player to act after the partner. Indeed, in (A)CG the player acting first has good chances to get the maximal payoff of *S* = 4 by insisting: when the second player knows that the partner insists, it is better to accommodate and get a payoff of *T* = 3, than to insist and get *R* = 2. In PD, however, defection is less beneficial if it can be discovered by the opponent and acted upon (for details, see Subsection “One-shot transparent Prisoner’s Dilemma with unequal reaction times” below). Therefore, in PD most players prefer acting later: defectors to have a better chance of getting *T* = 5 for a successful defection, and cooperators to make sure that the partners are not defecting them. The only exception from this rule is the Leader-Follower strategy, but as we show in S1 Note this special case does not change the overall situation for the simulations. Therefore, the optimal behaviour in PD is generally to wait as long as possible, while in (A)CG a player should act as quickly as possible. Consequently, when the time for making choice is bounded from below and from above, evolution in these games favours marginal mean reaction times: maximal allowed reaction time in PD and minimal allowed reaction time in (A)CG. Player types with different behaviour are easily invaded. Therefore we assumed in all simulations that the reaction times have a constant and equal mean. We also assumed that reaction times for all players have an equal non-zero variance and that the difference of the reaction time distributions for two types of players is always symmetric (see S1 Note). This results in $p_{\text{see}}^{i}$ being the same for all types, thus all players have equal chances to see the choices of each other.

### Analysis of one-shot transparent games

Consider a one-shot transparent game between Player 1 and Player 2 having strategies $s^{1}=\left(s_{1}^{1};s_{2}^{1};s_{3}^{1}\right)$ and $s^{2}=\left(s_{1}^{2};s_{2}^{2};s_{3}^{2}\right)$, and probabilities to see the choice of the partner $p_{\text{see}}^{1}$ and $p_{\text{see}}^{2}$, respectively. An expected payoff for Player 1 is given by

$$\begin{array}{cc} E(s^{1},s^{2}) & =\left(1-p_{\text{see}}^{1}-p_{\text{see}}^{2}\right)(s_{1}^{1}s_{1}^{2}R+s_{1}^{1}\left(1-s_{1}^{2}\right)S+\left(1-s_{1}^{1}\right)s_{1}^{2}T+\left(1-s_{1}^{1}\right)\left(1-s_{1}^{2}\right)P)\\ & +p_{\text{see}}^{2}s_{1}^{1}(s_{2}^{2}R+\left(1-s_{2}^{2}\right)S)+p_{\text{see}}^{2}\left(1-s_{1}^{1}\right)(s_{3}^{2}T+\left(1-s_{3}^{2}\right)P)\\ & +p_{\text{see}}^{1}s_{1}^{2}(s_{2}^{1}R+\left(1-s_{2}^{1}\right)T)+p_{\text{see}}^{1}\left(1-s_{1}^{2}\right)(s_{3}^{1}S+\left(1-s_{3}^{1}\right)P), \end{array}$$ (1)

where the first line describes the case when neither player sees partner’s choice, the second line describes the case when Player 2 sees the choice of Player 1, and the third—when Player 1 sees the choice of Player 2.

Let us provide two definitions that will be used throughout this section.

**Definition 1**. Strategies **s**^1^ and **s**^2^ are said to form a *Nash Equilibrium* if neither player would benefit from unilaterally switching to another strategy, that is E(**s**^1^, **s**^2^) ≥ E(**r**^1^, **s**^2^) and E(**s**^2^, **s**^1^) ≥ E(**r**^2^, **s**^1^) for any alternative strategies **r**^1^ and **r**^2^ of Players 1 and 2, respectively.

**Definition 2**. Let us denote E_*ij*_ = E(**s**^*i*^, **s**^*j*^). Strategy **s**^1^ is said to *dominate* strategy **s**^2^ if E_11_ ≥ E_21_ and E_12_ ≥ E_22_. If both inequalities are strict, **s**^1^ *strictly dominates* **s**^2^. Strategies **s**^1^ and **s**^2^ are said to be *bistable* when E_11_ > E_21_ and E_12_ < E_22_. Strategies **s**^1^ and **s**^2^ *co-exist* when E_11_ < E_21_ and E_12_ > E_22_.

Some intuition on these notions is provided below in subsection “Evolutionary dynamics of two strategies”. We refer to [9] for details.

For the sake of simplicity, we assume for the rest of this section that $0<p_{\text{see}}^{1},p_{\text{see}}^{2}<1$, otherwise the game is equivalent to the classic sequential or simultaneous game. First we consider the one-shot transparent Prisoner’s dilemma (PD), and then—(Anti-)Coordination Game ((A)CG).

#### One-shot transparent Prisoner’s Dilemma with equal reaction times

Here we assume that $p_{\text{see}}^{1}=p_{\text{see}}^{2}=p_{\text{see}}$ to simplify the discussion. Similar to the classic one-shot PD, in the transparent PD all Nash Equilibria (NE) correspond to mutual defection. To show this we make an important observation: in the one-shot PD it is never profitable to cooperate when seeing the partner’s choice.

**Lemma 1**. *In one-shot transparent PD with p*_see_ > 0 *any strategy* (*s*_1_; *s*_2_; *s*_3_) *with s*_2_, *s*_3_ > 0 *is dominated by strategies* (*s*_1_; 0; *s*_3_) *and* (*s*_1_; *s*_2_; 0). *The dominance of* (*s*_1_; 0; *s*_3_) *is strict when s*_1_ > 0, *the dominance of* (*s*_1_; *s*_2_; 0) *is strict when s*_1_ < 1.

*Proof*. Consider the strategies **s**^1^ = (*s*_1_; *s*_2_; 0), **s**^2^ = (*s*_1_; *s*_2_; *s*_3_). To show that **s**^1^ dominates **s**^2^, it is sufficient to demonstrate that E_11_ − E_21_ ≥ 0 and E_12_ − E_22_ ≥ 0. Two following inequalities can be inferred from (1), from the fact that in PD *R* < *T* and from the assumptions that *p*_see_, *s*_1_, *s*_2_ > 0:

$$\begin{array}{c} E_{11}-E_{21}=p_{\text{see}}s_{1}T-p_{\text{see}}s_{1}(s_{2}R+\left(1-s_{2}\right)T)=p_{\text{see}}s_{1}s_{2}\left(T-R\right)\geq 0,\\ E_{12}-E_{22}=p_{\text{see}}s_{1}T-p_{\text{see}}s_{1}(s_{2}R+\left(1-s_{2}\right)T)=p_{\text{see}}s_{1}s_{2}\left(T-R\right)\geq 0. \end{array}$$

As one can easily see, both inequalities are strict for *s*_1_ > 0. The second part of the proof follows from *S* < *P* and (1), and is otherwise identical, therefore we omit it.

Now we can describe the NE strategies in transparent PD:

**Proposition 2**. *In one-shot transparent PD all the Nash Equilibria are comprised by pairs of strategies* (0; *x*; 0) *with* 0 ≤ *x* ≤ 1 *and*

$$\begin{array}{c} x\leq \frac{1-p_{\text{see}}}{p_{\text{see}}}\frac{P-S}{R-S}. \end{array}$$ (2)

*Proof*. First we show that for any *x*, *y* satisfying (2), strategies (0; *x*; 0) and (0; *y*; 0) form a Nash Equilibrium. Assume that there exists a strategy (*s*_1_; *s*_2_; *s*_3_), which provides a better payoff against (0; *x*; 0) than (0; *y*; 0). According to Lemma 1, expected payoff of a strategy (*s*_1_; 0; 0) is not less than the payoff of (*s*_1_; *s*_2_; *s*_3_). Now it remains to find the value of *s*_1_ maximizing the expected payoff E of (*s*_1_; 0; 0). From (1) we have:

$$\begin{array}{cc} E & =\left(1-2p_{\text{see}}\right)(s_{1}S+\left(1-s_{1}\right)P)+p_{\text{see}}s_{1}\left(xR+\left(1-x\right)S\right)+p_{\text{see}}\left(1-s_{1}\right)P+p_{\text{see}}P\\ & =P+s_{1}(p_{\text{see}}x\left(R-S\right)-\left(1-p_{\text{see}}\right)\left(P-S\right))=P+s_{1}(x-\frac{1-p_{\text{see}}}{p_{\text{see}}}\frac{P-S}{R-S})p_{\text{see}}\left(R-S\right) \end{array}$$

Thus the expected payoff is maximized by *s*_1_ = 0 if inequality (2) holds and by *s*_1_ = 1 otherwise. In the former case the strategy (*s*_1_; 0; 0) results in the same payoff *P* as the strategy (0; *y*; 0), which proves that a pair of strategies (0; *x*; 0), (0; *y*; 0) is an NE. If (2) does not hold, strategy (0; *x*; 0) is not an NE, since switching to (1; 0; 0) results in a better payoff.

Let us show that there are no further NE. Indeed, according to Lemma 1 if an alternative NE exists, it can only consist of strategies (1; 0; *z*) or (*u*; 0; 0) with 0 ≤ *z* ≤ 1 and 0 < *u* < 1. In both cases switching to unconditional defection is preferable, which finishes the proof.

The one-shot transparent PD has two important differences from the classic game. First, the unconditional defection (0; 0; 0) dominates the cooperative strategy (1; 1; 0) only for $p_{\text{see}}<\frac{T-R}{T-P}$. Indeed, when both players stick to (1; 1, 0), their payoff is equal to *R*, while when switching to (0; 0; 0) strategy, a player gets *p*_see_*P* + (1 − *p*_see_)*T*. However, (1; 1, 0) is dominated by a strategy (1; 0; 0) that cooperates when it does not see the choice of the partner and defects otherwise. This strategy, in turn is dominated by (0; 0; 0).

Second, in transparent PD unconditional defection (0; 0; 0) is not evolutionary stable as players can switch to (0; *x*; 0) with *x* > 0 retaining the same payoff. This, together with Proposition 3 below, makes possible a kind of evolutionary cycle: (1; 0; 0) → (0; 0; 0) ↔ (0; *x*; 0) → (1; 1; 0), (1; 0; 0) → (1; 0; 0).

**Proposition 3**. *In transparent PD strategies* (1; 0; 0) *and* (0; *x*; 0) *have the following relations*:

1. *if condition* (2) *and the following condition*
  $$\begin{array}{c} x\leq \frac{1}{p_{\text{see}}}-2+\frac{P-S}{T-R} \end{array}$$ (3)
  *are satisfied, then* (0; *x*; 0) *dominates* (1; 0; 0);
2. *if neither* (2) *nor* (3) *are satisfied, then* (1; 0; 0) *dominates* (0; *x*; 0);
3. *if* (2) *is satisfied but* (3) *is not, then the two strategies coexist*;
4. *if* (3) *is satisfied but* (2) *is not, then the two strategies are bistable*.

*Proof*. We prove only the first statement since the proof of the others is almost the same.

Let Player 1 use strategy (1; 0; 0) and Player 2—strategy (0; *x*; 0). To prove that (0; *x*; 0) dominates (1; 0; 0) we need to show that Player 2 has no incentive to switch to (1; 0; 0) and that Player 1, on the contrary, would get higher payoff if using (0; *x*; 0). The latter statement follows from Proposition 2. To show that the former also takes place we simply write down expected payoffs E_11_ and E_21_ of strategies (1; 0; 0) and (0; *x*; 0) when playing against (1; 0; 0):

$$\begin{array}{c} E_{11}=\left(1-2p_{\text{see}}\right)R+p_{\text{see}}T+p_{\text{see}}S=R+p_{\text{see}}\left(T-2R\right)+p_{\text{see}}S,\\ E_{21}=\left(1-2p_{\text{see}}\right)T+p_{\text{see}}(xR+(1-x)T)+p_{\text{see}}P=T-p_{\text{see}}T+p_{\text{see}}P-xp_{\text{see}}\left(T-R\right). \end{array}$$

Now it can be easily seen that E_11_ ≤ E_21_ holds whenever inequality (3) is satisfied.

#### One-shot transparent Prisoner’s Dilemma with unequal reaction times

Here we consider the case when players have unequal probabilities to see partner’s choice. We focus on a simple example showing why waiting is generally beneficial in the transparent iPD. Assume that all players in population act as quickly as they can, but cooperation takes on average longer than defection. Assume further that a player preparing to cooperate may see the partner defecting and then it is still possible for this player to change decision and defect. Finally let us consider only pure strategies that is *s*_1_, *s*_2_, *s*_3_ ∈ {0, 1}. The question now is, which strategy would win in this case.

From Lemma 1, we know that it is sufficient to consider two strategies: “cooperators” **s**^1^ = (1; 0; 0) and “defectors” **s**^2^ = (0; 0; 0) since they dominate all other strategies. Note that the probability $p_{\text{see}}^{12}$ of cooperative players to see the choice of defectors is higher than the probability $p_{\text{see}}^{21}$ of defectors to see the choice of cooperators, resulting in $0<p_{\text{see}}^{21}<p_{\text{see}}^{12}<1$. Probabilities of a player to see the choice of another player with the same strategy is not higher than 0.5 (since these probabilities are equal for both players and the sum of these probabilities is not higher than 1), therefore it holds $0<p_{\text{see}}^{11},p_{\text{see}}^{22}\leq 0.5$. Note that, as before, we assume $0<p_{\text{see}}^{11},p_{\text{see}}^{12},p_{\text{see}}^{21},p_{\text{see}}^{22}<1$.

Then the expected payoff matrix for these two strategies in the one-shot transparent PD is given by

$$\begin{array}{cc} E & =(\begin{array}{cc} \left(1-2p_{\text{see}}^{11}\right)R+p_{\text{see}}^{11}\left(S+T\right) & p_{\text{see}}^{12}P+\left(1-p_{\text{see}}^{12}\right)S\\ p_{\text{see}}^{12}P+\left(1-p_{\text{see}}^{12}\right)T & P \end{array})\\ & =(\begin{array}{cc} R-p_{\text{see}}^{11}\left(2R-S-T\right) & p_{\text{see}}^{12}P+\left(1-p_{\text{see}}^{12}\right)S\\ T-p_{\text{see}}^{12}\left(T-P\right) & P \end{array}). \end{array}$$

Since $p_{\text{see}}^{12}P+\left(1-p_{\text{see}}^{12}\right)S<P$ for $p_{\text{see}}^{12}<1$, three variants are possible:

1. cooperative strategy **s**^1^ dominates for $p_{\text{see}}^{12}=1$;
2. **s**^1^ and **s**^2^ are bistable for E_11_ > E_21_, that is for
  $$\begin{array}{c} p_{\text{see}}^{12}>\frac{T-R}{T-P}+\frac{2R-S-T}{T-P}p_{\text{see}}^{11}; \end{array}$$ (4)
3. defecting strategy **s**^2^ dominates otherwise.

For the standard Prisoner’s Dilemma payoff matrix (Fig 2), inequality (4) turns into $p_{\text{see}}^{12}>\frac{1}{2}+\frac{1}{4}p_{\text{see}}^{11}$. Since $p_{\text{see}}^{11}\leq 0.5$, cooperative strategy **s**^1^ acting with a delay has a chance to win over defectors if it can see their actions with probability $p_{\text{see}}^{12}>5/8$. This example demonstrates that cooperation can survive in one-shot Prisoner’s dilemma under certain (artificial) assumptions. More importantly, this example shows the importance of seeing partner’s choice in transparent Prisoner’s Dilemma in general, illustrating the incentive of players to wait for partner’s action.

#### One-shot transparent (Anti-)Coordination Game

Recall [79] that in the classic one-shot (A)CG game there are three Nash Equilibria: two pure (Player 1 insists, Player 2 accommodates, or vice verse) and one mixed (each player insists with probability $\frac{S-P}{S+T-P-R}$). The Nash Equilibria for the transparent (A)CG game are specified by the following proposition.

**Proposition 4**. *Consider one-shot transparent (A)CG between Players 1 and 2 with probabilities to see the choice of the partner* $p_{\text{see}}^{12}$ and $p_{\text{see}}^{21}$, *respectively*. *Let* $p_{\text{see}}^{12}\leq p_{\text{see}}^{21}$, *then this game has the following pure strategy NE*.

1. *Player 1 uses strategy* (0; 0; 1), *Player 2 uses strategy* (1; 0; 1)—*for*
  $$\begin{array}{c} \frac{p_{\text{see}}^{21}}{1-p_{\text{see}}^{12}}\leq \frac{T-R}{S-R}; \end{array}$$ (5)
2. *Player 1 uses strategy* (1; 0; 1), *Player 2 uses strategy* (0; 0; 1)—*for*
  $$\begin{array}{c} \frac{p_{\text{see}}^{12}}{1-p_{\text{see}}^{21}}\leq \frac{T-R}{S-R} \end{array}$$ (6)
3. *Both players use strategy* (1; 0; 1)—*when* (6) *is not satisfied*.

*Additionally, if inequality* (5) *is satisfied, there is also a mixed-strategy NE: Player i uses strategy* $\left(s_{1}^{i};0;1\right)$ *with*

$$\begin{array}{c} s_{1}^{1}=\frac{\left(1-p_{\text{see}}^{21}\right)\left(S-P\right)-p_{\text{see}}^{12}\left(T-P\right)}{\left(1-p_{\text{see}}^{12}-p_{\text{see}}^{21}\right)\left(T+S-P-R\right)},\;\;s_{1}^{2}=\frac{\left(1-p_{\text{see}}^{12}\right)\left(S-P\right)-p_{\text{see}}^{21}\left(T-P\right)}{\left(1-p_{\text{see}}^{12}-p_{\text{see}}^{21}\right)\left(T+S-P-R\right)}. \end{array}$$ (7)

*In other words, when* (5) *holds, there are two pure-strategy and one mixed-strategy NE. Otherwise there is only one pure-strategy NE: Player 1 uses strategy* (1; 0; 1), *Player 2 uses strategy* (0; 0; 1) *when* (6) *holds, and both Players use* (1; 0; 1) *when* (6) *does not hold*.

*Remark* 1. For the correct interpretation of Proposition 4 it is important that inequality (6) holds automatically whenever (5) holds, since

$$\begin{array}{c} \frac{p_{\text{see}}^{12}}{1-p_{\text{see}}^{21}}\leq \frac{p_{\text{see}}^{21}}{1-p_{\text{see}}^{12}}. \end{array}$$

The latter statement follows from $p_{\text{see}}^{12}-p_{\text{see}}^{21}\leq 0$ (assumption of Proposition 4) and the fact that $1-p_{\text{see}}^{12}-p_{\text{see}}^{21}\geq 0$. Indeed, it holds $\left(p_{\text{see}}^{12}-p_{\text{see}}^{21}\right)\left(1-p_{\text{see}}^{12}-p_{\text{see}}^{21}\right)\leq 0$, and, consequently,

$$\begin{array}{c} p_{\text{see}}^{12}\left(1-p_{\text{see}}^{12}\right)\leq p_{\text{see}}^{21}\left(1-p_{\text{see}}^{12}-p_{\text{see}}^{21}\right)+p_{\text{see}}^{12}p_{\text{see}}^{21}=p_{\text{see}}^{21}\left(1-p_{\text{see}}^{21}\right). \end{array}$$

To prove Proposition 4, we need two lemmas. First, similar to the Prisoner’s dilemma, for the transparent (A)CG we have:

**Lemma 5**. *In one-shot transparent (A)CG any strategy* (*s*_1_; *s*_2_; *s*_3_) *is dominated by strategies* (*s*_1_; 0; *s*_3_) *and* (*s*_1_; *s*_2_; 1). *The dominance of* (*s*_1_; 0; *s*_3_) *is strict when s*_1_ > 0, *the dominance of* (*s*_1_; *s*_2_; 1) *is strict when s*_1_ < 1.

The proof is based on the fact that for the (A)CG game hold inequalities *R* < *T* and *P* < *S*. Otherwise the proof is identical to the proof of Lemma 1.

**Lemma 6**. *In one-shot transparent (A)CG, when Player 1 uses strategy* (1; 0; 1), *the best response for Player 2 is to use strategy* (0; 0; 1) *for* $\frac{p_{\text{see}}^{12}}{1-p_{\text{see}}^{21}}\leq \frac{T-R}{S-R}$ *and to use* (1; 0; 1) *otherwise*.

*Proof*. By Lemma 5 the best response for Player 2 is a strategy (*s*_1_; 0; 1) with 0 ≤ *s*_1_ ≤ 1. When Player 2 uses this strategy against (1; 0; 1), the expected payoff of Player 2 is given by

$$\begin{array}{cc} E_{21} & =\left(1-p_{\text{see}}^{12}-p_{\text{see}}^{21}\right)(s_{1}R+\left(1-s_{1}\right)T)+p_{\text{see}}^{12}(s_{1}S+\left(1-s_{1}\right)T)+p_{\text{see}}^{21}T\\ & =T+s_{1}(p_{\text{see}}^{12}\left(S-R\right)+p_{\text{see}}^{21}\left(T-R\right)-\left(T-R\right)). \end{array}$$

Thus the payoff of Player 2 depends linearly on the value of *s*_1_ and is maximized by *s*_1_ = 0 if

$$\begin{array}{c} p_{\text{see}}^{12}\left(S-R\right)-\left(1-p_{\text{see}}^{21}\right)\left(T-R\right)<0 \end{array}$$ (8)

and by *s*_1_ = 1 otherwise. Inequality (8) is equivalent to (6), which completes the proof.

Using Lemmas 5 and 6, we can now compute NE for the one-shot transparent (A)CG:

*Proof*. Pure strategy NEs are obtained immediately from Lemma 6. To compute the mixed-strategy NE, recall that Player 1 achieves it when the expected payoff obtained by Player 2 for insisting and accommodating is equal:

$$\begin{array}{c} \left(1-p_{\text{see}}^{12}-p_{\text{see}}^{21}\right)\left(s_{1}^{1}R+\left(1-s_{1}^{1}\right)S\right)+p_{\text{see}}^{12}S=\left(1-p_{\text{see}}^{12}-p_{\text{see}}^{21}\right)\left(s_{1}^{1}T+\left(1-s_{1}^{1}\right)P\right)+p_{\text{see}}^{12}T. \end{array}$$

By computing $s_{1}^{1}$ from this equation and applying the same argument for Player 2, we get the strategy entries given in (7).

**Corollary 7**. *Consider one-shot transparent (A)CG with S* = 4, *T* = 3, *R* = 2, *P* = 1, *where both players have equal probabilities p*_see_ *to see the choice of the partner. In this game there are three NE for p*_see_ < 1/3: *(a) Player 1 uses strategy* (1; 0; 1), *Player 2 uses strategy* (0; 0; 1); *(b) vice versa; (c) both players use strategy* (*x*; 0; 1), *with* $x=\frac{3}{4}+\frac{p_{\text{see}}}{4-8p_{\text{see}}}$. *For p*_see_ ≥ 1/3, (1; 0; 1) *is the only NE*.

#### One-shot transparent Hawk-Dove and Leader games

While we consider in Proposition 4 only the (A)CG game for which *S* > *T* > *R* > *P*, this result is easily generalized to a wider class of (anti-)coordination games including several other important games, such as Hawk-Dove and Leader. Together with PD and (A)CG, Hawk-Dove and Leader form the set of two-player two-action games where players have a conflict of interests [29]. Hawk-Dove (also known as Chicken or Snowdrift) game is also relevant for studying the evolution of cooperation, competition over a shared resource, and reciprocity [61, 80–82]. Note that in [83], (A)CG, Leader and Hawk-Dove are grouped into a single category category (category III) that is referred to as “Hawk-Dove” games. This categorization is based on equilibrium structure of the simultaneous game: two strict asymmetric NE and one symmetric mixed NE, which is evolutionary stable strategy. This structure is not by default the same for the transparent games, but the following result takes place.

**Proposition 8**. *Consider a general one-shot transparent game between Players 1 and 2 with probabilities to see the choice of the partner* $p_{\text{see}}^{12}$ and $p_{\text{see}}^{21}$ *satisfying* $p_{\text{see}}^{12}\leq p_{\text{see}}^{21}$. *If the payoff matrix of the game satisfies inequalities S* > *T* > *R and S* > *P*, *then the Nash equilibria (NE) of this game is described by Proposition 4. Namely, when* (5) *holds, there are two pure-strategy and one mixed-strategy NE. Otherwise there is only one pure-strategy NE: Player 1 uses strategy* (1; 0; 1), *Player 2 uses strategy* (0; 0; 1) *when* (6) *holds, and both Players use* (1; 0; 1) *when* (6) *does not hold*.

*Proof*. The proof coincides with the proof of Proposition 4. Indeed, Lemma 5 holds for any game with *T* > *R* and *S* > *P*. Lemma 6 holds whenever *T* > *R* and *S* > *R*. Finally, NE are described in Proposition 4 for the case *S* > *T*.

Two classical games satisfy conditions of Proposition 8. The first is the “Hawk-Dove” game, where *S* > *P* > *T* > *R*. To get the classic notation for this game, one needs to replace in Fig 2B “Insist” by “Hawk” and “Accommodate” by “Dove”.

The second relevant game is Leader, described by *S* > *T* > *P* > *R*. This game is similar to the (A)CG game formulated as insisting on own preference or accommodating the other, with the difference that here if both players insist it is detrimental for both, so it is better to accommodate, however the insisting player receives maximal reward if the other player accommodates. An example of a Leader game payoff matrix can be obtained from that in Fig 2B by setting *P* = 2, *R* = 1, while leaving *S* = 4, *T* = 3.

The payoff matrices of these games are illustrated in the S2 Fig.

### Analysis of iterated transparent games

For the analysis of iterated games we use the techniques described in [9, 24]. Since most of results for simultaneous and sequential iPD were obtained for strategies taking into account outcomes of the last interaction (“memory-one strategies”), here we also focus on memory-one strategies. Note that considering multiple previous round results in very complex strategies. To overcome this, one can, for instance, use pure strategies (see, for instance, [31]), but we reserve this possibility for future research.

A strategy without memory in transparent games is described by a three-element vector. A memory-one strategy additionally conditions current choice upon the outcome of the previous round of the game. Since there are 4 = 2 × 2 possible outcomes, a memory-one strategy for a player type *i* is represented by a vector $s={\left(s_{k}^{i}\right)}_{k=1}^{12}$, where *k* enumerates the twelve (4 × 3) different combinations of previous outcome and the current probability of choice. The entries $s_{k}^{i}$ of the strategy thus represent the conditional probabilities to select action A_1_ (“Cooperate” in iPD and “Insist” in i(A)CG, see Fig 2), specifically

$s_{1}^{i},\ldots ,s_{4}^{i}$ are probabilities to select A_1_ without seeing the partner’s choice, given that in the previous round the joint choice of the player and the partner was A_1_A_1_, A_1_A_2_, A_2_A_1_, and A_2_A_2_ respectively (the first action specifies the choice of the player, and the second—the choice of the partner);
$s_{5}^{i},\ldots ,s_{8}^{i}$ are probabilities to select A_1_, seeing the partner selecting A_1_ and given the outcome of the previous round (as before).
$s_{9}^{i},\ldots ,s_{12}^{i}$ are probabilities to select A_1_, seeing the partner selecting A_2_ and given the outcome of the previous round.

Probabilities to select A_2_ are given by $\left(1-s_{k}^{i}\right)$, respectively.

Consider an infinite population of players evolving in generations. For any generation *t* = 1, 2, … the population consists of *n*(*t*) player types defined by their strategies $s^{i}={\left(s_{k}^{i}\right)}_{k=1}^{12}$ and their frequencies *x*_*i*_(*t*) in the population, $\sum_{i=1}^{n(t)}x_{i}\left(t\right)=1$. Besides, the probability of a player from type *i* to see the choice of a partner from type *j* is given by $p_{\text{see}}^{ij}\in \left[0,1\right]$ (in our case $p_{\text{see}}^{ij}=p_{\text{see}}$ for all types *i* and *j*, but in this section we use the general notation).

Consider a player from type *i* playing an infinitely long iterated game against a player from type *j*. Since both players use memory-one strategies, this game can be formalized as a Markov chain with states being the mutual choices of the two players and a transition matrix *M* given by

$$\begin{array}{c} M=\left(1-p_{\text{see}}^{ij}-p_{\text{see}}^{ji}\right)M_{0}+p_{\text{see}}^{ij}M_{1}+p_{\text{see}}^{ji}M_{2}, \end{array}$$ (9)

where the matrices *M*_0_, *M*_1_ and *M*_2_ describe the cases when neither player sees the choice of the partner, Player 1 sees the choice of the partner before making own choice, and Player 2 sees the choice of the partner, respectively. These matrices are given by

$$\begin{array}{c} M_{0}=(\begin{array}{cccc} s_{1}^{i}s_{1}^{j} & s_{1}^{i}\left(1-s_{1}^{j}\right) & \left(1-s_{1}^{i}\right)s_{1}^{j} & \left(1-s_{1}^{i}\right)\left(1-s_{1}^{j}\right)\\ s_{2}^{i}s_{3}^{j} & s_{2}^{i}\left(1-s_{3}^{j}\right) & \left(1-s_{2}^{i}\right)s_{3}^{j} & \left(1-s_{2}^{i}\right)\left(1-s_{3}^{j}\right)\\ s_{3}^{i}s_{2}^{j} & s_{3}^{i}\left(1-s_{2}^{j}\right) & \left(1-s_{3}^{i}\right)s_{2}^{j} & \left(1-s_{3}^{i}\right)\left(1-s_{2}^{j}\right)\\ s_{4}^{i}s_{4}^{j} & s_{4}^{i}\left(1-s_{4}^{j}\right) & \left(1-s_{4}^{i}\right)s_{4}^{j} & \left(1-s_{4}^{i}\right)\left(1-s_{4}^{j}\right) \end{array}), \end{array}$$

$$\begin{array}{c} M_{1}=(\begin{array}{cccc} s_{5}^{i}s_{1}^{j} & s_{9}^{i}\left(1-s_{1}^{j}\right) & \left(1-s_{5}^{i}\right)s_{1}^{j} & \left(1-s_{9}^{i}\right)\left(1-s_{1}^{j}\right)\\ s_{6}^{i}s_{3}^{j} & s_{10}^{i}\left(1-s_{3}^{j}\right) & \left(1-s_{6}^{i}\right)s_{3}^{j} & \left(1-s_{10}^{i}\right)\left(1-s_{3}^{j}\right)\\ s_{7}^{i}s_{2}^{j} & s_{11}^{i}\left(1-s_{2}^{j}\right) & \left(1-s_{7}^{i}\right)s_{2}^{j} & \left(1-s_{11}^{i}\right)\left(1-s_{2}^{j}\right)\\ s_{8}^{i}s_{4}^{j} & s_{12}^{i}\left(1-s_{4}^{j}\right) & \left(1-s_{8}^{i}\right)s_{4}^{j} & \left(1-s_{12}^{i}\right)\left(1-s_{4}^{j}\right) \end{array}), \end{array}$$

$$\begin{array}{c} M_{2}=(\begin{array}{cccc} s_{1}^{i}s_{5}^{j} & s_{1}^{i}\left(1-s_{5}^{j}\right) & \left(1-s_{1}^{i}\right)s_{9}^{j} & \left(1-s_{1}^{i}\right)\left(1-s_{9}^{j}\right)\\ s_{2}^{i}s_{7}^{j} & s_{2}^{i}\left(1-s_{7}^{j}\right) & \left(1-s_{2}^{i}\right)s_{11}^{j} & \left(1-s_{2}^{i}\right)\left(1-s_{11}^{j}\right)\\ s_{3}^{i}s_{6}^{j} & s_{3}^{i}\left(1-s_{6}^{j}\right) & \left(1-s_{3}^{i}\right)s_{10}^{j} & \left(1-s_{3}^{i}\right)\left(1-s_{10}^{j}\right)\\ s_{4}^{i}s_{8}^{j} & s_{4}^{i}\left(1-s_{8}^{j}\right) & \left(1-s_{4}^{i}\right)s_{12}^{j} & \left(1-s_{4}^{i}\right)\left(1-s_{12}^{j}\right) \end{array}). \end{array}$$

The gain of type *i* when playing against type *j* is given by the expected payoff E_*ij*_, defined by

$$\begin{array}{c} E_{ij}=y_{R}R+y_{S}S+y_{T}T+y_{P}P, \end{array}$$ (10)

where *R*, *S*, *T*, *P* are the entries of the payoff matrix (*R* = 3, *S* = 0, *T* = 5, *P* = 1 for standard iPD and *R* = 2, *S* = 4, *T* = 3, *P* = 1 for i(A)CG, see Fig 2), and *y*_*R*_, *y*_*S*_, *y*_*T*_, *y*_*P*_ represent the probabilities of getting to the states associated with the corresponding payoffs by playing **s**^*i*^ against **s**^*j*^. This vector is computed as a unique left-hand eigenvector of matrix *M* associated with eigenvalue one [9]:

$$\begin{array}{c} \left(y_{R},y_{S},y_{T},y_{P}\right)=\left(y_{R},y_{S},y_{T},y_{P}\right)M. \end{array}$$

The evolutionary success of type *i* is encoded by its fitness *f*_*i*_(*t*): if type *i* has higher fitness than the average fitness of the population $\bar{f}\left(t\right)=\sum_{i=1}^{n(t)}x_{i}\left(t\right)f_{i}\left(t\right)$, then *x*_*i*_(*t*) increases with time, otherwise *x*_*i*_(*t*) decreases and the type is dying out. This evolutionary process is formalized by the replicator dynamics equation, which in discrete time takes the form

$$\begin{array}{c} x_{i}\left(t+1\right)=x_{i}\left(t\right)+\frac{f_{i}\left(t\right)-\bar{f}\left(t\right)}{\bar{f}\left(t\right)}x_{i}\left(t\right)=\frac{f_{i}\left(t\right)}{\bar{f}\left(t\right)}x_{i}\left(t\right). \end{array}$$ (11)

The fitness *f*_*i*_(*t*) is computed as the average payoff for a player of type *i* when playing against the current population:

$$\begin{array}{c} f_{i}\left(t\right)=\sum_{j=1}^{n(t)}x_{j}\left(t\right)E_{ij}, \end{array}$$

where E_*ij*_ is given by (10).

#### Evolutionary dynamics of two strategies

To provide an example of evolutionary dynamics and introduce some useful notation, we consider a population consisting of two types playing iPD with strategies: **s**^1^ = (1, 0, 0, 1; 1, 0, 0, 1; 0, 0, 0, 0), **s**^2^ = (0, 0, 0, 0; 0, 0, 0, 0; 0, 0, 0, 0) (recall that we write 0 instead of *ε* and 1 instead of 1 − *ε* for *ε* = 0.001; see Results, section Transparent games with memory: evolutionary simulations) and initial conditions *x*_1_(1) = *x*_2_(1) = 0.5. That is, the first type plays the “Win–stay, lose–shift” (WSLS) strategy, and the second type (almost) always defects (uses the AllD strategy). We set $p_{\text{see}}^{11}=p_{\text{see}}^{12}=p_{\text{see}}^{21}=p_{\text{see}}^{22}=p_{\text{see}}$. Note that since $p_{\text{see}}^{11},p_{\text{see}}^{22}\leq 0.5$ and $p_{\text{see}}^{12}+p_{\text{see}}^{21}\leq 1$, it holds *p*_see_ ≤ 0.5. Given *p*_see_ we can compute a transition matrix of the game using (9) and then calculate the expected payoffs for all possible pairs of players *ij* using (10). For instance, for *p*_see_ = 0 and *ε* = 0.001 we have

$$\begin{array}{c} E_{11}=2.995,E_{12}=0.504,E_{21}=2.999,E_{22}=1.003. \end{array}$$

This means that a player of the WSLS-type on average gets a payoff E_11_ = 2.995 when playing against a partner of the same type, and only E_12_ = 0.504, when playing against an AllD-player. The fitness for each type is given by

$$\begin{array}{c} f_{1}\left(t\right)=x_{1}\left(t\right)E_{11}+x_{2}\left(t\right)E_{12}=2.995x_{1}\left(t\right)+0.504x_{2}\left(t\right),\\ f_{2}\left(t\right)=x_{1}\left(t\right)E_{21}+x_{2}\left(t\right)E_{22}=2.999x_{1}\left(t\right)+1.003x_{2}\left(t\right). \end{array}$$

**Fig 9:**
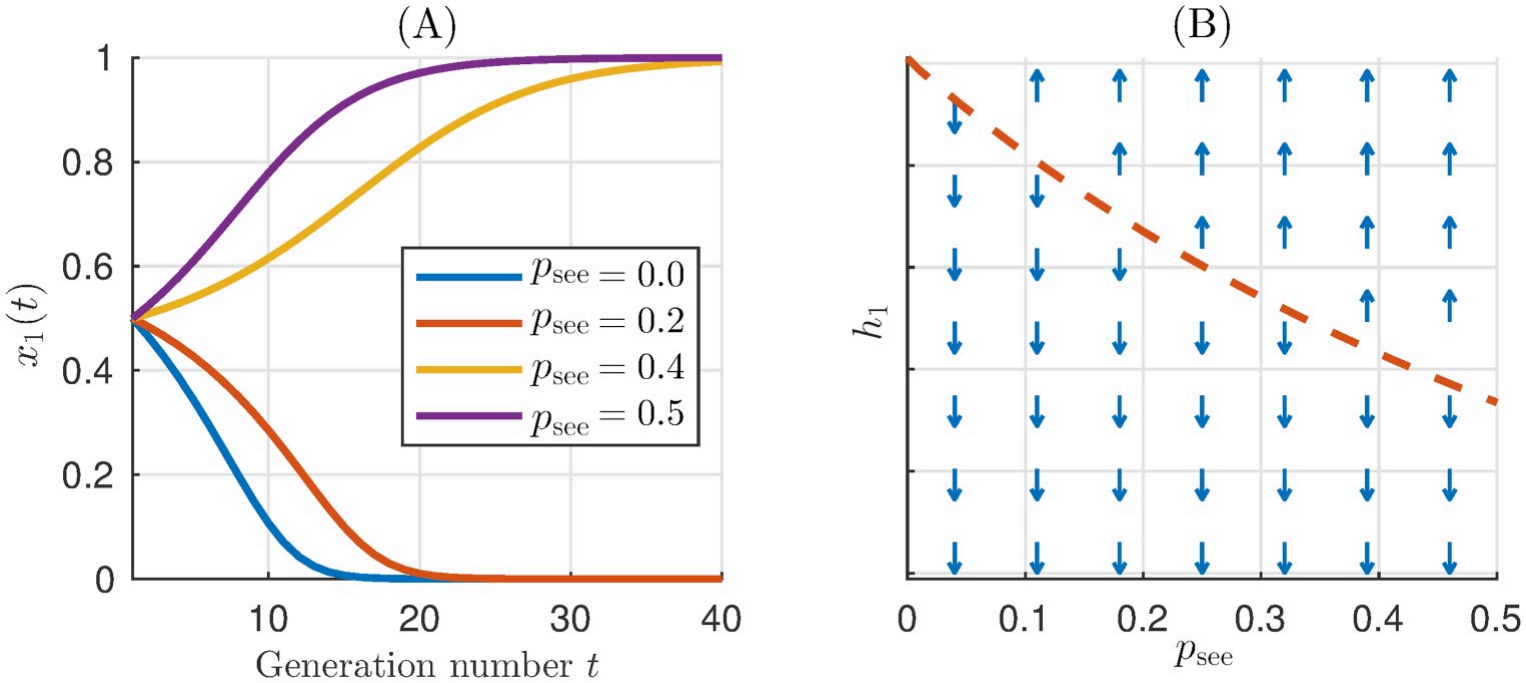
Evolutionary dynamics of iPD-population consisting of two types of players: With WSLS and AllD strategies. (A) Initially, both types have the same frequency, but after 40 generations the fraction of WSLS-players *x*_1_(*t*) converges to 0 for probabilities to see partner’s choice *p*_see_ = 0.0, 0.2 and to 1 for *p*_see_ = 0.4, 0.5. (B) This is due to the decrease of the invasion threshold *h*_1_ for WSLS: while *h*_1_ = 1 for *p*_see_ = 0 (AllD dominates WSLS and the fraction of WSLS-players unconditionally decreases), AllD and WSLS are bistable for *p*_see_ > 0 and WSLS wins whenever *x*_1_(*t*)>*h*_1_. Arrows indicate whether frequency *x*_1_(*t*) of WSLS increases or decreases. Interestingly, *h*_1_ = 0.5 holds for *p*_see_ ≈ 1/3, which corresponds to the maximal uncertainty since the three cases (“Player 1 knows the choice of Player 2 before making its own choice”; “Player 2 knows the choice of Player 1 before making its own choice”; “Neither of players knows the choice of the partner”) have equal probabilities.

Since *f*_2_(*t*) > *f*_1_(*t*) for any 0 < *x*_1_(*t*), *x*_2_(*t*) < 1, the AllD-players take over the whole population after several generations. Dynamics of the type frequencies *x*_*i*_(*t*) computed using (11) shows that this is indeed the case (Fig 9A). Note that since E_21_ > E_11_ and E_22_ > E_12_, AllD is garanteed to win over WSLS for any initial frequency of WSLS-players *x*_1_(1). In this case one says that AllD *dominates* WSLS and can *invade* it for any *x*_1_(1).

As we increase *p*_see_, the population dynamics changes. While for *p*_see_ = 0.2 AllD still takes over the population, for *p*_see_ = 0.4 WSLS wins (Fig 9A). This can be explained by computing the expected payoff for *p*_see_ = 0.4:

$$\begin{array}{c} E_{11}=2.995,E_{12}=0.628,E_{21}=2.500,E_{22}=1.003. \end{array}$$

Hence *f*_1_(*t*) > *f*_2_(*t*) for 0 ≤ *x*_2_(*t*) ≤ 0.5 ≤ *x*_1_(*t*) ≤ 0, which explains the observed dynamics. Note that here E_11_ > E_21_, while E_12_ < E_22_, that is when playing with WSLS- and AllD-players alike partners of the same type win more than partners of a different type. In this case one says that WSLS and AllD are *bistable* and there is an unstable equilibrium fraction of WSLS players given by

$$\begin{array}{c} h_{1}=\frac{E_{22}-E_{12}}{E_{11}-E_{12}-E_{21}+E_{22}}. \end{array}$$ (12)

We call *h*_*i*_ an *invasion threshold* for type *i*, since this type takes over the whole population for *x*_*i*_(*t*) > *h*_*i*_, but dies out for *x*_*i*_(*t*) < *h*_*i*_. To illustrate this concept, we plot in Fig 9A the invasion threshold *h*_1_ as a function of *p*_see_ for WSLS type playing against AllD.

The third possible case of two-types dynamics is *coexistence*, which takes place when E_11_ < E_21_, E_12_ > E_22_, that is when playing against a player of any type is less beneficial for a partner of the same type than for a partner of a different type. In this case the fraction of a type given by (12) corresponds to a stable equilibrium meaning that the frequency of the first type *x*_1_(*t*) increases for *x*_1_(*t*) < *h*_1_, but decreases for *x*_1_(*t*) > *h*_1_.

#### Evolutionary simulations for transparent games

Theoretical analysis of the strategies in repeated transparent games is complicated due to the many dimensions of the strategy space, which motivates using of evolutionary simulations. For this we adopt the methods described in [9, 24]. We do not use here a more modern adaptive dynamics approach [84, 85] since for high-dimensional strategy space it would require analysis of a system with many equations, complicating the understanding and interpretation of the results.

Each run of simulations starts with five player types having equal initial frequencies: *n*(1) = 5, *x*_1_(1) = … = *x*_5_(1) = 0.2. Following [24], strategy entries $s_{k}^{i}$ with *k* = 1, …, 12 for each player type *i* are randomly drawn from the distribution with U-shaped probability density, favouring probability values around 0 and 1:

$$\begin{array}{c} \rho \left(y\right)=\pi (y(1-y){)}^{-1/2} \end{array}$$ (13)

for *y* ∈ (0, 1). Additionally, we require $s_{k}^{i}\in \left[\varepsilon ,1-\varepsilon \right]$, where *ε* = 0.001 accounts for the minimal possible error in the strategies [24]. The fact that players cannot have pure strategies and are prone to errors is also closely related to the “trembling hand” effect preventing players from using pure strategies [24, 86]. We performed evolutionary simulations for various transparencies with *p*_see_ = 0.0, 0.1, …, 0.5.

The frequencies of strategies *x*_*i*_(*t*) change according to the replicator Eq (11). If *x*_*i*_(*t*) < *χ*, the type is assumed to die out and is removed from the population (share *x*_*i*_(*t*) is distributed proportionally among the remaining types); we follow [9, 24] in taking *χ* = 0.001. Occasionally (every 100 generations on average to avoid strong synchronization), new types are entered in the population. The strategies for the new types are drawn from (13) and the initial frequencies are set to *x*_*i*_(*t*_0_) = 1.1*χ* [24].

## Supporting information

S1 Note Transparent games and reaction times distributions. (PDF)

S2 Note Transparent iterated Prisoner’s Dilemma with a restricted strategy space. (PDF)

S1 Fig Distributions of total shares in the population over all generations for 80 most persistent player types over the 80 runs of evolutionary simulations. (A) for iterated Prisoner’s Dilemma (iPD) and (B) for iterated (Anti-)Coordination Game (i(A)CG). The central mark indicates the median, and the bottom and top edges of the box indicate the 25th and 75th percentiles, respectively. The whiskers extend to the most extreme data points not considered outliers, and the outliers are plotted individually using the ‘+’ symbol. The higher total shares of the types are, the more stable the dynamics in the population is. While stability varies with transparency for both games, the drop of stability in iPD for *p*_see_ ≥ 0.4 is especially noticeable. Indeed, in highly transparent iPD any strategy is sufficiently “predictable”, which allows a best-response strategy to replace it in a population. Such best-response strategies can be generally weak and short-living, see for example treacherous WSLS described in Fig 5 (main text). Note that stability increases considerably for *p*_see_ ≥ 0.4 in i(A)CG, which reflects the fact that Leader-Follower strategy becomes evolutionary stable for high transparency.
(TIF)

S2 Fig Generalized payoff matrix of (anti-)coordination games and its particular cases: (A)CG, Leader, and Hawk-Dove, expressed as ordinal payoffs. (TIF)
