# Supplementary material for: Emergence and suppression of cooperation by action visibility in transparent games
Source: PLoS Comput Biol. 2020 Jan 9;16(1):e1007588. doi: 10.1371/journal.pcbi.1007588 (PMC6975562; doi:10.1371/journal.pcbi.1007588)
Supplement: S1 Note — (PDF) [file pcbi.1007588.s001.pdf]

Emergence and suppression of cooperation by action visibility in transparent games: Supplementary note 1

Anton M. Unakafov   Thomas Schultze   Alexander Gail   Sebastian Moeller
Igor Kagan   Stephan Eule   Fred Wolf

Transparent games and reaction times distributions

In the Methods section we argue that evolution favours equal reaction times both in iPD and i(A)CG, since the optimal behaviour in iPD is to wait as long as possible, and in i(A)CG – to act as quickly as possible. However, for iPD there is a notable exception: the Leader-Follower (L-F) strategy is better off when acting fast and exposing its choice to the partner. Consider, for instance, a population consisting of L-F players of two types, the first acting fast and the second waiting. In all inter-type interactions, players of the first type have an upper hand since they take the role of Leaders, maximizing own payoff. Thus the first type dominates the second and finally takes over the population. The question then is, whether this contradiction to the general rule for the transparent iPD (to wait as long as possible) changes the simulation results?

Additional simulations show that this is not the case. We have used the same evolutionary simulations as before with one modification. Instead of using for all types a fixed probability to see the partner's choice p_{see}, we computed this probability for each pair of types as shown in SN1 Fig. 1: from the reaction times (RT) modelled by exponentially modified Gaussian distributions and from the visibility threshold ΔT.

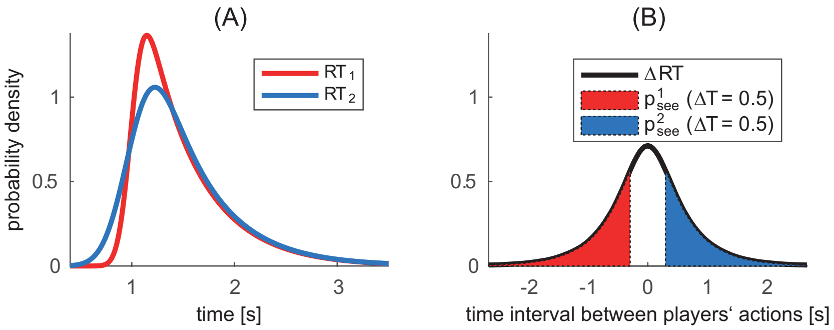

SN1 Figure 1: **Distributions of reaction times (RT) of the players determine their probability to see the partner's choice.** (A) RT distributions for two players modelled by exponentially modified Gaussian distribution. (B) Distribution of RT difference $\Delta\text{RT} = \text{RT}_2 - \text{RT}_1$ and probabilities to see the partner's choice given by $p^{12}_{\text{see}} = \Pr(\Delta\text{RT} < -\Delta T)$ (Player 1 knows the choice of Player 2 before making own choice, the blue area) and $p^{21}_{\text{see}} = \Pr(\Delta\text{RT} > \Delta T)$ (vice versa, the red area), where ΔT is a time interval required for a player to interpret and act on the partner's choice, and Pr stands for the probability mapping.

Exponentially modified Gaussian distribution has three parameters: mean of Gaussian component μ, standard deviation of Gaussian component σ and relaxation time of exponential component τ. For each type of players a random mean reaction time μ was selected from the set $\{2.0, 2.1, \ldots, 3.0\}$. Since we were mainly interested in the influence of the types' mean RT on the results, we set other parameters to constants: $\sigma = 0.1$ and $\tau = 0.5$.

For each two types i and j we computed probabilities to see partner's choice as follows:

1. Using exponentially modified Gaussian distribution, we generated for each type samples of reaction times $\text{RT}_{i,k}$, $\text{RT}_{j,k}$ for $k = 1, 2, \ldots, K$ with $K = 10^6$.

2. We computed reaction time differences between types i and j by $\Delta \mathrm{RT}_k = \mathrm{RT}_{j,k} - \mathrm{RT}_{i,k}$.

3. We estimated probabilities to see partner's choice by

$$p^{ij}_{\text{see}} = \frac{1}{K}\sharp\{k = 1, \dots, K \mid \Delta \mathrm{RT}_k < -\Delta T\},$$
$$p^{ji}_{\text{see}} = \frac{1}{K}\sharp\{k = 1, \dots, K \mid \Delta \mathrm{RT}_k > \Delta T\},$$

where $\sharp A$ stands for the number of elements in the set A.

We performed three series of evolutionary simulations for $\Delta T = 1.98,\ 0.478,\ 0.001$. These values were selected so that for any type i probability p^{ii}_{see} was equal to 0.001, 0.2 and 0.499, respectively. Each series consisted of 80 runs of evolutionary simulations, we traced 10^9 generations in each run. Except the way the values of p^{ij}_{see} were computed, the simulations were as described in the main text of the manuscript

As expected, results were similar to those with equal RT but more noisy since additional type variability increases the number of generations necessary for the population to reach the equilibrium state. In SN1 Fig. 2A, for low (but non-zero) transparency WSLS wins with a total relative frequency above 85% (without GWSLS), but as transparency increases the share of WSLS drops down. On the contrary, the Leader-Follower strategy has the best performance for high transparency with a relative frequency 27% (SN1 Fig. 2C). Note that all successful types have marginal RT: WSLS-players mostly have maximal reaction times, while L-F-players have minimal reaction times.

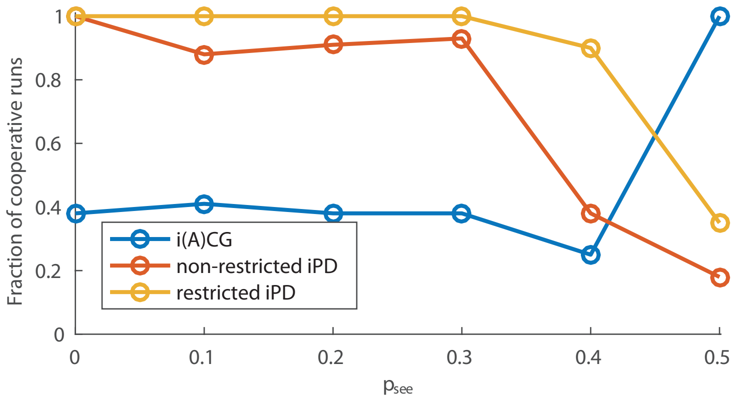

SN1 Figure 2: **Fractions of the most frequent strategies in transparent iPD with unequal reaction times (RT).** RT were modelled by exponentially modified Gaussian distributions with μ randomly selected from the set $\{2.0, 2.1, \dots, 3.0\}$, $\sigma = 0.1$ and $\tau = 0.5$. WSLS is considered here together with GWSLS, they have a strategy profile ($1abc$;1***;****) with $a, b < 2/3$, $c \geq 2/3$. We characterized as L-F all strategies with a profile (*$00b$;****;*$11c$), where $b < 1/3$ and $c < 2/3$. Finally, we considered a strategy as defecting if it has entries $s_4, s_{12} < 0.2$, $s_1, s_2, s_3 < 1/3$ and $s_8 < 2/3$. (A) For low transparencies WSLS is predominate and WSLS-players clearly prefer waiting over fast action. (B) For moderate transparencies population is controlled either by the waiting WSLS players or by the fast-acting defectors, though the latter are successful only since many strategies may have $s^i_9, s^i_{10}, s^i_{11}, s^i_{12} > 0$, resulting in cooperation with apparent defectors. (C) For high transparencies Leader-Follower outperforms defecting strategies. Note that in all cases types with marginal RT prevail and the observed strategy frequencies are similar to those for equal RT.

The only principal difference from the simulations with fixed p_{see} takes place for moderate transparencies, in particular, for $\Delta T = 0.478$ when probability to see the partner's choice in intra-type interactions is given by $p^{ii}_{\text{see}} = 0.2$. SN1 Fig. 2B shows that in this case defecting strategies have an unexpectedly high relative frequency. However, this seems to be an artefact caused by the fact that for the most types added to the population strategy entries $s^i_9, s^i_{10}, s^i_{11}, s^i_{12} > 0$ (meaning that players may cooperate even seeing that partner defects). Playing against fast-acting defectors, these types take the

role of Followers and become an easy prey. Indeed, if a defecting strategy has $\mu_i = 2$, its opponent with $\mu_j = 2.5$ sees the choice of the defector with probability $p^{ji}_{\text{see}} > 0.5$, and an opponent with $\mu_j = 3$ with probability $p^{ji}_{\text{see}} > 0.8$. In this case probabilities $s^i_9, \ldots, s^i_{12}$ are much more important than for the case when RT are equal and these entries are used only with probability $p^{ii}_{\text{see}} = 0.2$. Fast-acting defecting strategies can be only counteracted by TFT-like strategies with $s^i_9, s^i_{10}, s^i_{11}, s^i_{12} \approx 0$. Note that the L-F strategy is not successful against defecting strategies in this case, since L-F can only survive for high p^{ii}_{see}.
