# Supplementary material for: Emergence and suppression of cooperation by action visibility in transparent games
Source: PLoS Comput Biol. 2020 Jan 9;16(1):e1007588. doi: 10.1371/journal.pcbi.1007588 (PMC6975562; doi:10.1371/journal.pcbi.1007588)
Supplement: S2 Note — (PDF) [file pcbi.1007588.s002.pdf]

Emergence and suppression of cooperation by action visibility in transparent games: Supplementary note 2

Anton M. Unakafov Thomas Schultze Alexander Gail Sebastian Moeller
Igor Kagan Stephan Eule Fred Wolf

Transparent iterated Prisoner's dilemma with a restricted strategy space

Here we introduce a variant of transparent iterated Prisoner's dilemma (iPD) with a restricted strategy space. Note that in iPD a rational player in most cases would not cooperate seeing that partner defects. The only notable exception is the Leader-Follower strategy. In general, one can see in Figure 4 (main text) that probabilities $s_9, \ldots, s_{12}$ to cooperate seeing that partner defects are quite low, especially for $p_{\text{see}} < 0.4$ (note that this takes place despite of the fact that defection for $p_{\text{see}} < 0.4$ is rare, meaning that entries $s_9, \ldots, s_{12}$ are not very important for the strategy success).

Assuming that cooperation with a defecting partner is unnatural, we can set $s_9 = \ldots = s_{12} = 0$. A question then is, whether such priors change the dynamics of the iPD-strategies. SN2 Fig. 1 shows that restricting strategy space results in the same drop of cooperation as in the non-restricted iPD.

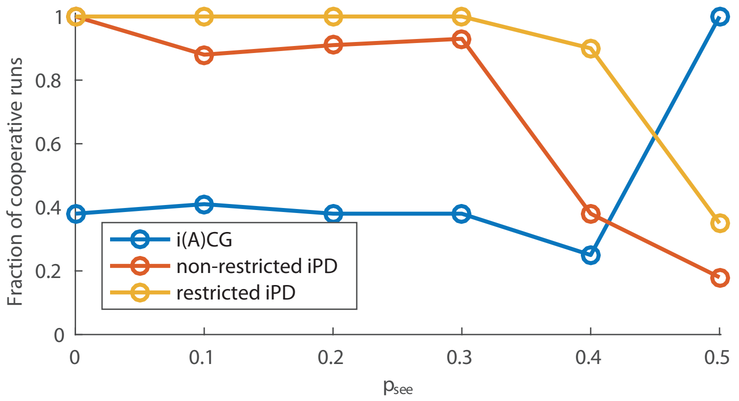

SN2 Figure 1: **Fraction of runs for which cooperation was established in iterated (Anti-) Coordination Game (i(A)CG) and in two versions of iterated Prisoner's Dilemma (iPD).** We assumed that cooperation was established in the population if the average payoff was above $0.9 \cdot 3$ for iPD and above $0.95 \cdot 3.5$ for i(A)CG (90% and 95% of maximal possible value). In iPD (for both non-restricted and restricted strategy space) seeing the partner's choice adversely affects cooperation as it increases the temptation to exploit the partner. In i(A)CG, "evolution" results in more cooperative agents when they have a higher probability of seeing the partner's choice as this helps them to coordinate. The small drop in cooperation for i(A)CG at $p_{\text{see}} = 0.4$ is caused by a transition from turn-taking to leader-following.

There is however, one difference: SN2 Fig. 2 shows that for high p_{see} an "inverse Leader-Follower" strategy (inverse L-F) emerges instead of Leader-Follower introduced for the non-restricted iPD. Inverse L-F is theoretically represented by $\mathbf{s} = (1110; 0000; 0000)$, that is the player cooperates when it does not see the choice of the partner and defects otherwise. In the simultaneous iPD ($p_{\text{see}} = 0$) L-F behaves as unconditional cooperator and is easily beaten, but it becomes predominant in restricted settings for $p_{\text{see}} = 0.5$. Note that inverse L-F is an extension of the strategy $(1; 0; 0)$, which plays a special role in one-shot PD (see "Methods" section). However, memory provides to inverse L-F an important

advantage: it can distinguish unconditional defectors AllD from conspecifics. Resistance to AllD is achieved by defecting after mutual defection ($s_4 = 0$).

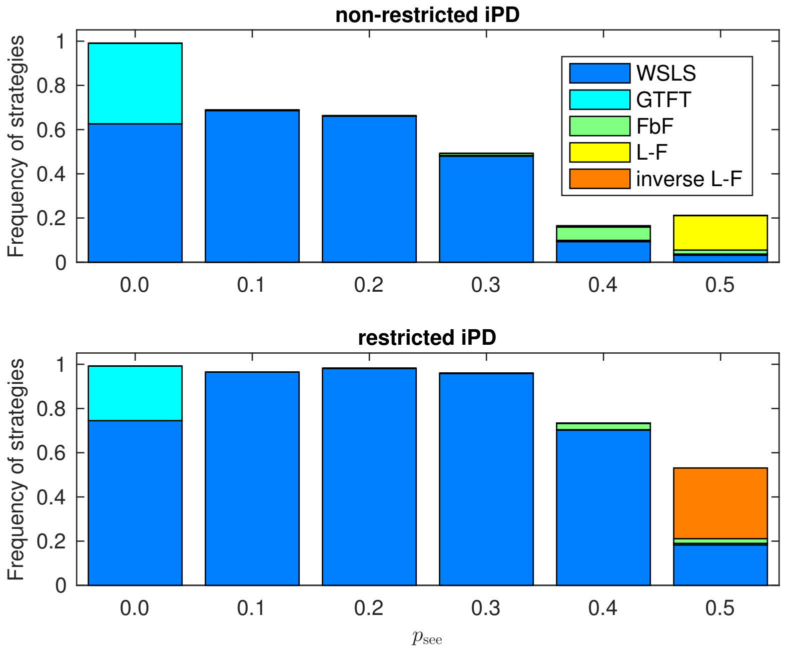

SN2 Figure 2: **Fractions of WSLS, GTFT, FbF and L-F strategies in transparent iPD with non-restricted and restricted strategy space.** The frequencies were computed over 10^9 generations in 80 runs. Note the striking similarities between two scenarios. The main differences include the lower stability in the non-restricted iPD and emergence of inverse L-F instead of L-F for $p_{\text{see}} = 0.5$ in restricted iPD. We classified as inverse L-F all strategies with profile (*11*; *00*; 0000) since behaviour after mutual cooperation or mutual defection is only relevant when inverse L-F is playing against another strategy, and success for different types of behaviour depends on the composition of the population.

Spread of inverse L-F in the restricted iPD for high transparency illustrates pervasiveness of "Leader-Follower" principle. It also shows that the role of initiators can vary: in some cases, these agents reap special benefits, but in other cases they also carry the burden. Although counter-intuitive at first glance, the cooperativeness of Leaders in the L-F strategy corresponds to the behaviour of individuals that agree to do a necessary but risky or unpleasant job without immediate benefit. Examples include volunteering in human societies and acting as sentries in animal groups.
