# Supplementary figures and images for: Emergence and suppression of cooperation by action visibility in transparent games
Source: PLoS Comput Biol. 2020 Jan 9;16(1):e1007588. doi: 10.1371/journal.pcbi.1007588 (PMC6975562; doi:10.1371/journal.pcbi.1007588)

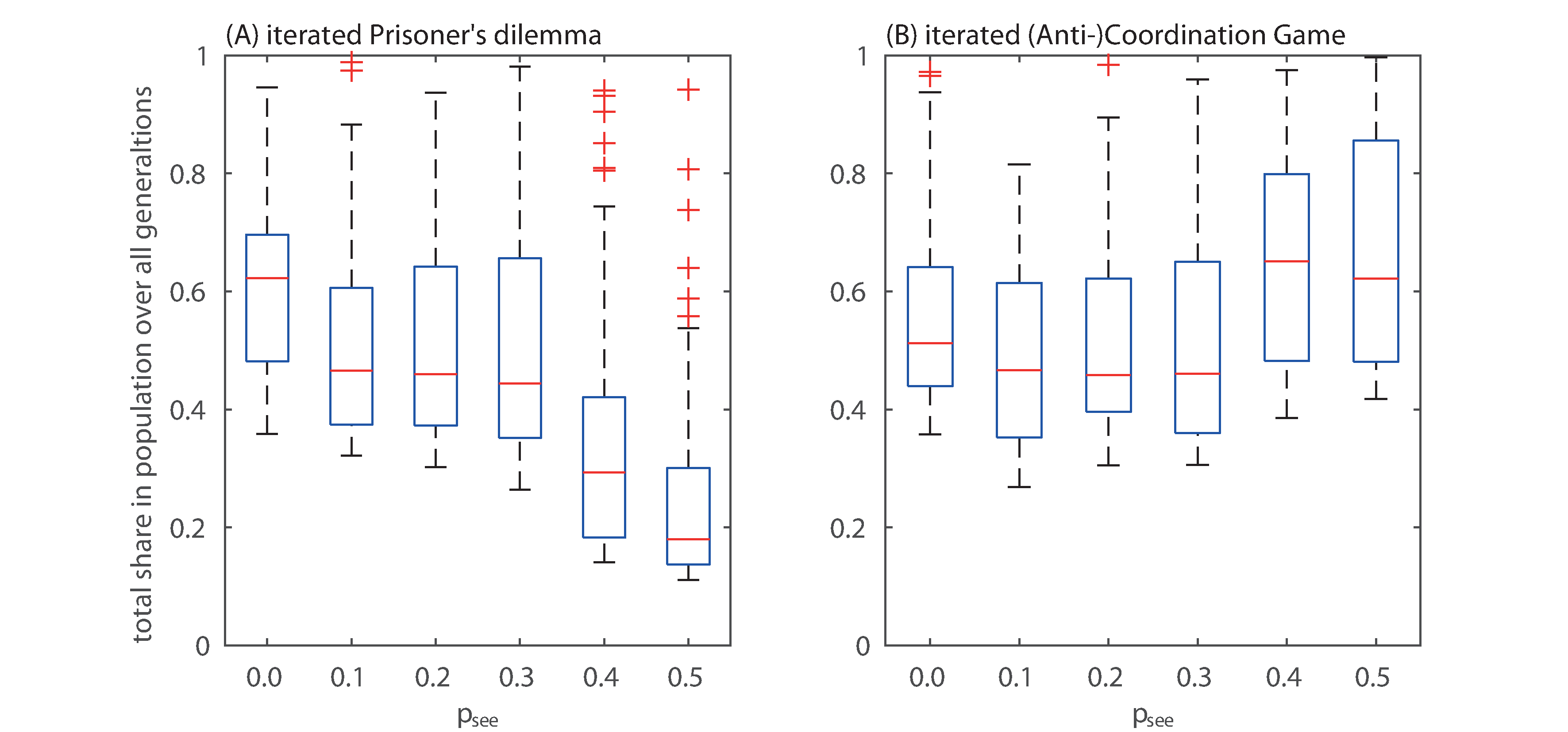

Supplement: S1 Fig — (A) for iterated Prisoner’s Dilemma (iPD) and (B) for iterated (Anti-)Coordination Game (i(A)CG). The central mark indicates the median, and the bottom and top edges of the box indicate the 25th and 75th percentiles, respectively. The whiskers extend to the most extreme data points not considered outliers, and the outliers are plotted individually using the ‘+’ symbol. The higher total shares of the types are, the more stable the dynamics in the population is. While stability varies with transparency for both games, the drop of stability in iPD for psee ≥ 0.4 is especially noticeable. Indeed, in highly transparent iPD any strategy is sufficiently “predictable”, which allows a best-response strategy to replace it in a population. Such best-response strategies can be generally weak and short-living, see for example treacherous WSLS described in Fig 5 (main text). Note that stability increases considerably for psee ≥ 0.4 in i(A)CG, which reflects the fact that Leader-Follower strategy becomes evolutionary stable for high transparency. (TIF) [file pcbi.1007588.s003.tif]

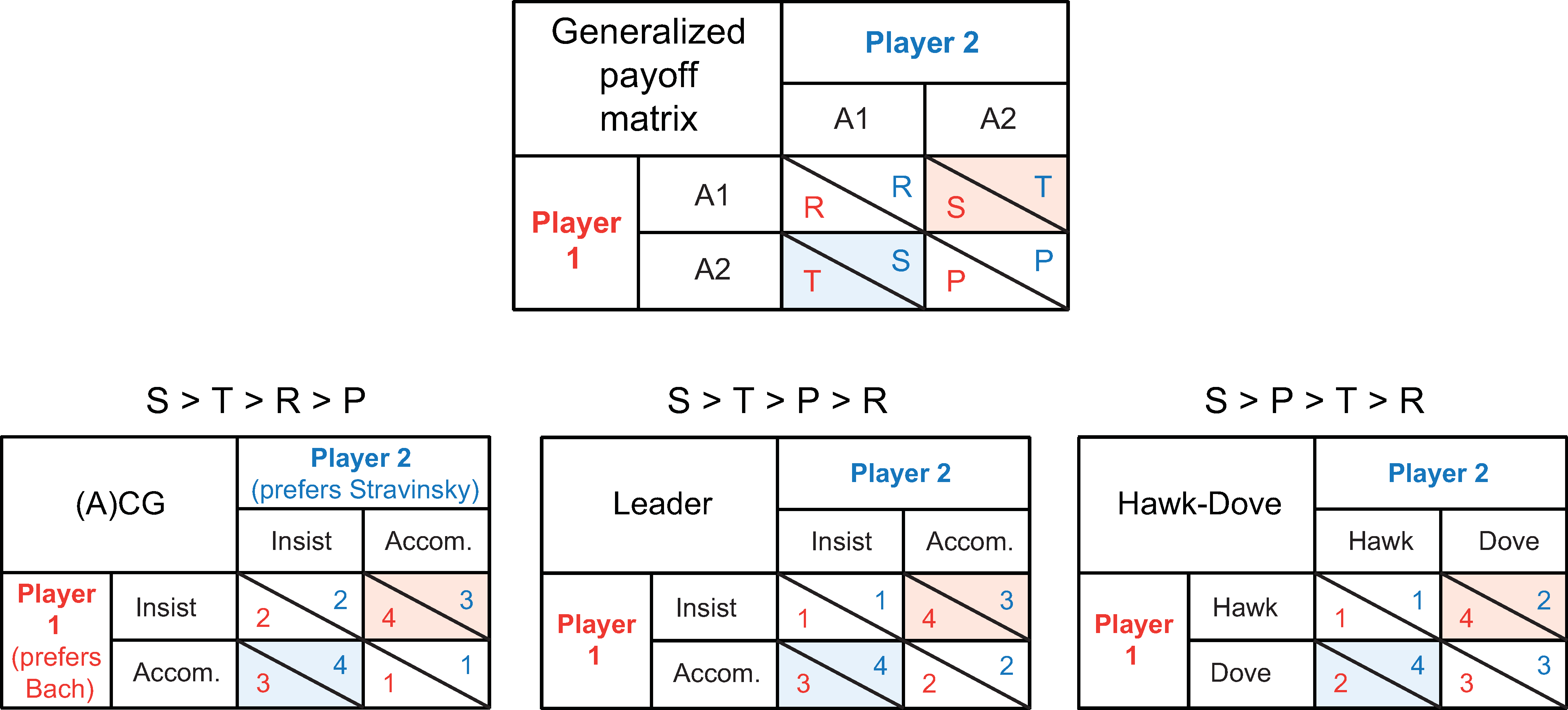

Supplement: S2 Fig — (TIF) [file pcbi.1007588.s004.tif]
